# Metabolomics Signatures of Exposure to Ambient Air Pollution: A Large-Scale Metabolome-Wide Association Study in the Cancer Prevention Study-II Nutrition Cohort

**DOI:** 10.1021/acs.est.4c09592

**Published:** 2024-12-16

**Authors:** Donghai Liang, Ziyin Tang, W. Ryan Diver, Jeremy A. Sarnat, Sabrina S. Chow, Haoran Cheng, Emily L. Deubler, Youran Tan, Stephanie M. Eick, Michael Jerrett, Michelle C. Turner, Ying Wang

**Affiliations:** † Gangarosa Department of Environmental Health, Rollins School of Public Health, Emory University, 1518 Clifton Road, Atlanta, Georgia 30322, United States; ‡ Department of Population Science, American Cancer Society, 270 Peachtree Street NW, Suite 1300, Atlanta, Georgia 30303, United States; § Department of Environmental Health Sciences, Fielding School of Public Health, University of California Los Angeles, Los Angeles, California 90095, United States; ∥ Barcelona Institute for Global Health (ISGlobal), Barcelona 08036, Spain; ⊥ Universitat Pompeu Fabra (UPF), Barcelona 08018, Spain; # CIBER Epidemiología y Salud Pública (CIBERESP), Madrid 28029, Spain

**Keywords:** air pollution, mixture, high-resolution metabolomics, metabolome-wide association study, oxidative stress, inflammation

## Abstract

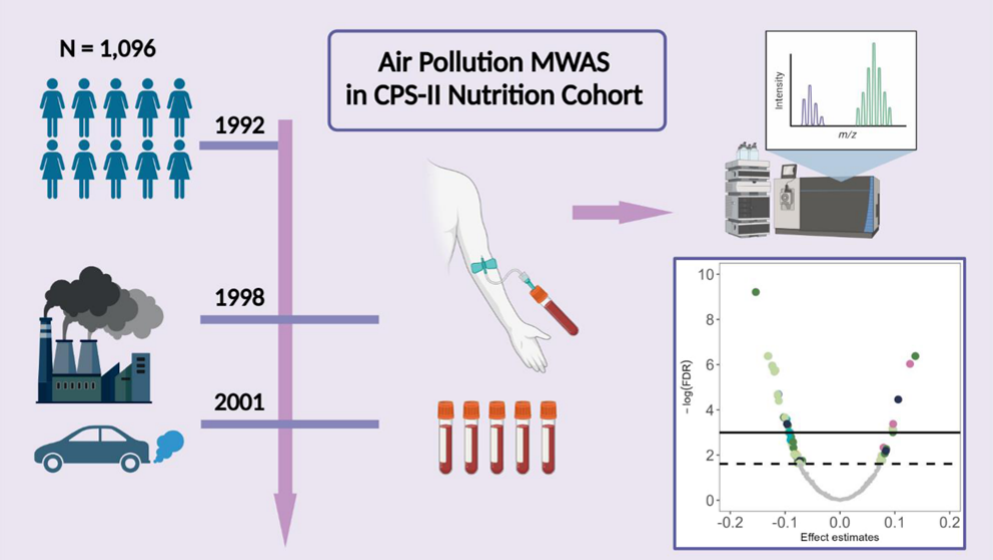

Existing air pollution metabolomics studies showed inconsistent results, often limited by small sample size and individual air pollutants effects. We conducted a metabolome-wide association study among 1096 women (68.2 ± 5.7 years) who provided blood samples (1998–2001) within the Cancer Prevention Study-II Nutrition Cohort. Annual average individual exposures to particulate matter, nitrogen dioxide, ozone, sulfur dioxide, and carbon monoxide in the year of blood draw were used. Metabolomics profiling was conducted on serum samples by Metabolon. We evaluated the individual air pollutants effects using multiple linear regression and the mixture effect using quantile g-computation, adjusting for confounders and false discovery rate (FDR). Ninety-five metabolites were significantly associated with at least one air pollutant or mixture (FDR < 0.05). These metabolites were enriched in pathways related to oxidative stress, systemic inflammation, energy metabolism, signals transduction, nucleic acid damage and repair, and xenobiotics. Sixty metabolites were confirmed with level 1 or 2 evidence, among which 21 have been previously linked to air pollution exposure, including taurine, creatinine, and sebacate. Overall, our results replicate prior findings in a large sample and provide novel insights into biological responses to long-term air pollution exposure using mixture analysis.

## Abstract 2

This large cross-sectional analysis of individual air pollutant effects and overall mixture effect on serum metabolites provides novel insights into understanding mechanisms underlying air pollution-related adverse health responses.

## Introduction

The associations between exposure to ambient air pollution and a range of adverse health outcomes are well established.^1−5^ Findings from the Cancer Prevention Study (CPS)-II cohort have contributed substantially to the scientific evidence associating increasing levels of specific air pollutants with higher mortality from respiratory disease, cardiometabolic disease, and lung cancer.^6−9^ Despite these well-recognized health impacts of ambient air pollution, uncertainty remains regarding the specific biological pathways mediating observed responses,^10−13^ and how these potential mechanisms may lead to individual susceptibility.^1,2,4,5^ Detailed characterizations of internal biological responses are critical to further clarifying which specific mechanisms underlie ambient air pollution toxicity. This task is complicated given the lack of sensitive and specific air pollution exposure biomarkers to measure internal exposures and corresponding physiological responses.^14,15^

High-resolution metabolomics (HRM), an innovative analytical platform that couples high-resolution mass spectrometry with various chromatographic separation strategies, has emerged as a promising tool to identify air pollution-related biomarkers by identifying thousands of metabolic features associated with exogenous exposures and endogenous processes.^16−19^ We previously demonstrated the applicability of HRM in linking air pollution exposure and internal biological responses in several panel and cohort studies of specific subpopulations.^20−29^ Despite the growing interest in HRM applications involving air pollution and health,^30−33^ the field remains nascent, with ongoing questions concerning the coherence and generalizability of the findings across study cohorts and analytical platforms.^34^ One potential cause of the inconsistency is that most existing air pollution HRM studies were conducted in relatively small study settings (i.e., *N* < 200), which may result in increased risks of false positive findings likely due to insufficient statistical power.^22,24,35,36^ Second, while air pollution is a complex mixture consisting of heterogeneous and highly correlated components, few studies have examined the joint effects of air pollution mixtures on the human metabolome. Given that individuals are exposed to various air pollutants simultaneously, it is critical to obtain a better understanding on how air pollution mixtures collectively impact human metabolome. Finally, over 80% of the significant metabolic features previously reported to be associated with ambient air pollution cannot be annotated or verified, resulting in uncertainties and inconsistencies in the downstream biological pathway analyses. All of these challenges necessitate further research using HRM in larger, well-characterized general population cohorts, with consideration of overall air pollution mixture to understand the potential joint effects and enhanced chemical annotation processes to examine consistencies and provide additional verification.

To address these critical knowledge gaps, we conducted a large-scale cross-sectional metabolomics study to evaluate the individual and potential joint effects of multiple air pollutants on serum metabolome in 1096 women enrolled in the CPS-II Nutrition Cohort.^37^ We followed an established untargeted metabolome-wide association study (MWAS) framework to evaluate metabolites and their metabolic pathways associated with long-term exposure to ambient air pollution.^23^ Here, we present the MWAS results, compare and summarize the findings from both individual air pollutant models and overall air pollution mixture models, and evaluate the consistency of our findings with other studies examining the metabolic response to ambient air pollution exposure.

## Methods

### Study Design and Population

The CPS-II Cohort is a U.S. prospective cohort established by the American Cancer Society in 1982 and enrolled nearly 1.2 million participants in 50 states, the District of Columbia, and Puerto Rico.^38^ Participants in this study were drawn from the CPS-II Nutrition Cohort, a subset of the larger CPS-II Cohort established between 1992 and 1993. The CPS-II Nutrition Cohort included over 180,000 men and women aged 50–74 years residing in 21 U.S. states with high-quality population-based cancer registries. Participants completed a self-administered questionnaire at baseline including demographic, medical, lifestyle, and other information. 39,200 participants of the CPS-II Nutrition Cohort also provided nonfasting blood samples between 1998 and 2001 that were stored at a central repository for future analysis. The cohort and sample collection process are described in detail elsewhere.^39^ The study protocols were approved by the Emory University (Atlanta, GA) Institutional Review Board.

We retrieved data from 782 postmenopausal breast cancer cases and 782 matched controls in a previous nested case-control study on metabolomics and breast cancer risk within the CPS-II Nutrition Cohort. The cases included all instances of breast cancers that occurred among postmenopausal women who provided nonfasting blood samples from 1998 to 2001. All women were cancer-free (except nonmelanoma skin cancer) at the time of blood draw. The healthy controls were 1:1 matched to the cases by date of birth (±6 months), race/ethnicity (Caucasian, African American, or other/unknown), and time at blood draw (±6 months) by incidence density sampling. Details on the study design and population characteristics can be found elsewhere.^37^ Since metabolomics profiles were only available for this population, we included all participants initially.

For this air pollution metabolomics analysis, a set of exclusion criteria was applied (Figure S1). To reduce potential misclassification and enhance temporal alignment among residential history, blood sampling, and air pollution data, we excluded women without matchable residential air pollution data (*N* = 284), those with different residential states at enrollment and at the time of blood draw (*N* = 10), and those missing annual average air pollution exposure data in the year of blood draw (those provided a blood sample in 1998; *N* = 85). We also excluded women without complete covariate data for statistical analyses (*N* = 72). A total of 1096 women were included in the subsequent analyses.

### Retrospective Residential Air Pollution Assessment

We retrieved air pollution assessments for each participant based on the residential address from the Center for Clean Air Climate Solution (CACES) database.^40^ Briefly, ambient air pollution exposure for the contiguous U.S. was estimated using integrated empirical geographic regression models.^40^ The prediction models were based on land use regression, employing variable selection and data reduction techniques to include a set of geographic characteristics from measures of traffic, land use, land cover, and satellite-based estimates of air pollution. Residential addresses were collected in 1982 when CPS-II was established. The residential addresses were geocoded and were used to link with outdoor air pollution data at the census block group level. Detailed information on the geocoding of participant residences can be found elsewhere.^41^ We included six air pollutants, which were fine particulate matter (PM_2.5_), coarse particulate matter (PM_10_), nitrogen dioxide (NO_2_), ozone (O_3_), sulfur dioxide (SO_2_), and carbon monoxide (CO). Our study aims to examine associations between long-term air pollution exposure and serum metabolomics. Although multiple critical exposure time windows are plausible, there is currently no evidence indicating which window may hold greater significance.^29,42^ Since the CACES database provides only annual average exposure data, we selected annual average exposure levels as the exposure time window to align with both our research interests and data availability. The Pearson correlation among air pollutants was examined. A detailed description of the exposure assignment methodology and corresponding associations with mortality can be found elsewhere.^7−9,43^

### Metabolomics Profiling

Metabolomics profiling on serum samples was conducted by Metabolon, Inc. (Durham, NC) using ultrahigh-performance liquid chromatography-tandem mass spectrometry (UPLC-MS/MS) as described elsewhere.^44^ Briefly, the serum samples were treated with methanol to precipitate proteins. Four sample fractions were dried and reconstituted in different solvents for measurement using four different platforms: (1) two fractions were analyzed by two separate reverse-phase UPLC-MS/MS methods with positive-ion-mode electrospray ionization (ESI); (2) one fraction was analyzed by reverse-phase UPLC-MS/MS with negative-ion-mode ESI; and (3) one fraction was analyzed by hydrophilic interaction chromatography UPLC-MS/MS with negative-ion-mode ESI. Samples from each case and its matched control were measured within the same batch, with pairs randomly assigned across batches. Individual metabolites were identified by comparison with a chemical library consisting of >5400 commercially available purified standard compounds.

A total of 1384 metabolites were detected. Triplicates of 46 samples were used as quality control samples to evaluate the reproducibility of the platform. Any missing values were assigned the minimum detection value. We excluded metabolites with a detection rate below 10% of samples (*n* = 109) and metabolites with intraclass correlation coefficient (ICC) < 0.5 (n = 89). As a result, 1186 metabolites were included in the analysis. The median ICC was 0.91 with an IQR of 0.82–0.96, suggesting a very high reproducibility of the platform. The median between-batch coefficient of variation (CV%) was 17% with an IQR of 11–27%. To correct for day-to-day variation from the platform, account for non-normal distribution, and allow comparison on the same scale, the relative concentration of each metabolite (“intensity”) was divided by its daily median, then log-transformed followed by autoscaled.

### Covariates Selection and Definition

Based on the existing literature, data availability, and a directed acyclic graph (Figure S2), the selected covariates were age at blood draw (continuous), body mass index (BMI; continuous), diet score (continuous), race (categorical: white and nonwhite), smoking status (categorical: never, former, and current smoker), year of blood draw (categorical: 1999, 2000, and 2001), and hours since last meal (categorical: <2 h ago, 2–4 h ago, and >4 h ago). Race was collected at baseline in 1982. Age at blood draw, BMI, smoking status, and hours since the last meal were collected in the survey at the blood draw. The diet score (ranges from 0–9) was derived using food items reported in the 1999 survey based on the 2006 ACS guidelines on nutrition and physical activity for cancer prevention, previously described elsewhere.^45^ Briefly, the diet score was based on the consumption of a variety of vegetables and fruits, the percentage of grains consumed as whole grains, and the consumption of processed and red meats. A higher diet score indicates higher concordance with the ACS guideline.

### Statistical Analysis

We employed two approaches to investigate the association between air pollution exposure and the serum metabolome. First, we utilized multiple linear regression models to examine the impacts of individual air pollutants on each metabolite. Specially, the natural log-transformed standardized intensity of each metabolite was regressed on the annual average level of each air pollutant, adjusting for the selected covariates. The effect estimates were expressed as the percent change in standardized metabolite intensities per interquartile range (IQR) increase in air pollution exposure levels, controlling for covariates. For each air pollutant, the general form of models are expressed as
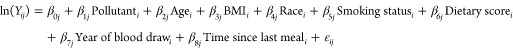
where *Y*_*ij*_ denotes the intensity of metabolites *j* for participants *i*. Pollutant_*i*_ refers to the annual average levels for participants *i.* ε_*ij*_ denotes residual random normal error.

Second, we applied quantile g-computation models to examine the overall effect of air pollution mixtures on each metabolite. Quantile g-computation provides a single effect estimate for an exposure mixture, simplifying interpretation and computational process without assuming directional homogeneity.^46^ Additionally, it provides a set of weights that describe the contribution of each exposure (positive or negative partial effect) to the overall effect estimate. The natural log-transformed standardized intensity of each metabolite was regressed on the annual average level of all air pollutants, adjusting for the selected covariates. The effect estimates were expressed as percent changes in standardized metabolite intensities per two quartiles (50%) increase in all air pollutant levels, controlling for covariates. The general models are expressed as
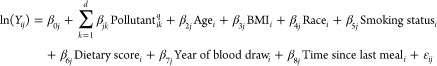
Where *Y*_*ij*_ denotes the intensity of metabolites *j* for participants *i*. *d* indicates the total number of air pollutants (*d* = 1, 2,..., 6). Pollutant_*ik*_^*q*^ refers to the quantiles of air pollutants *k* for participants *i.* ε_*ij*_ denotes residual random normal error.This analysis was conducted using the “*qgcomp.noboot*” function from the “*qgcomp*” package. To correct for multiple comparisons, we applied the Banjamini–Hochberg procedure, and a false discovery rate (FDR) < 0.05 is considered statistically significant. All analyses were conducted using R (version 4.1.0).

We conducted several sensitivity analyses. Specifically, we used the annual average level 1 year prior to the time of blood draw as exposure indicator (*N* = 772). To examine whether future breast cancer status would affect the results, we (1) further included breast cancer status (2-level factor, case, or control) in the models; and also (2) reran the analysis using control participants only (*N* = 528). We also ran analysis among never-smokers only (*N* = 606), to address potential residual confounding by smoking status of air pollution-metabolite associations.

## Results

A total of 1096 women were included in the analysis. The mean age and BMI were 68.2 ± 5.7 and 25.7 ± 4.6 kg/m^2^, respectively (Table 1). The majority (98%) of participants were white. Never-smokers, former-smokers, and current-smokers accounted for 55, 41, and 4%, respectively. Of all participants, 30, 65, and 6% contributed blood samples in 1999, 2000, and 2001, respectively. Participants were from 19 states, with the proportions of participants from each state ranging from 1.0 to 13.0% (Figure S3). The annual mean ± standard deviation concentrations of PM_2.5_, PM_10_, NO_2_, O_3_, SO_2_, and CO of the year of blood sample collection were 12.8 ± 3.1 μg/m^3^, 21.6 ± 6.5 μg/m^3^, 13.8 ± 5.9 ppb, 48.1 ± 7.0 ppb, 3.60 ± 1.8 ppb, and 0.48 ± 0.18 ppm, respectively (Table 2). The air pollutants were weakly to strongly correlated with one another (ρ = 0.06 for O_3_ and CO and ρ = 0.73 for PM_2.5_ and PM_10_) (Figure S4).

**Table 1:** Demographic Characteristics of the Study Population (*N* = 1096 women)^a^

		PM_2.5_		PM_10_		NO_2_		O_3_		SO_2_		CO	
	overall (*N* = 1096)	Q1 (*N* = 220)	Q5 (*N* = 219)	Q1 (*N* = 220)	Q5 (*N* = 219)	Q1 (*N* = 220)	Q5 (*N* = 219)	Q1 (*N* = 220)	Q5 (*N* = 219)	Q1 (*N* = 220)	Q5 (*N* = 219)	Q1 (*N* = 220)	Q5 (*N* = 219)
age (years), mean (SD)	68.2 (5.71)	68.5 (5.97)	68.2 (5.60)	67.9 (5.85)	68.7 (5.52)	68.2 (5.89)	68.7 (5.79)	68.4 (5.90)	68.0 (5.81)	68.2 (5.41)	67.8 (5.98)	67.9 (5.75)	68.6 (5.62)
BMI, mean (SD)	25.7 (4.59)	25.6 (4.35)	25.4 (4.59)	24.7 (3.88)	25.7 (4.88)	25.9 (4.49)	25.2 (4.45)	26.0 (4.59)	25.0 (4.47)	25.6 (4.65)	25.4 (4.67)	25.9 (4.32)	25.1 (4.40)
dietart score^b^, mean (SD)	4.34 (2.02)	4.30 (2.15)	4.47 (1.99)	4.49 (2.11)	4.33 (2.01)	4.37 (2.16)	4.37 (1.88)	4.25 (2.07)	4.23 (1.93)	4.40 (1.90)	4.29 (2.04)	4.33 (2.09)	4.39 (1.87)
race, *n* (%)													
White	1071 (97.7)	217 (98.6)	205 (93.6)	217 (98.6)	210 (95.9)	218 (99.1)	204 (93.2)	212 (96.4)	215 (98.2)	217 (98.6)	213 (97.3)	219 (99.5)	208 (95.0)
non-White	25 (2.3)	3 (1.4)	14 (6.4)	3 (1.4)	9 (4.1)	2 (0.9)	15 (6.8)	8 (3.6)	4 (1.8)	3 (1.4)	6 (2.7)	1 (0.5)	11 (5.0)
smoking status, *n* (%)													
never smoker	606 (55.3)	123 (55.9)	118 (53.9)	115 (52.3)	134 (61.2)	131 (59.5)	111 (50.7)	118 (53.6)	109 (49.8)	127 (57.7)	103 (47.0)	138 (62.7)	124 (56.6)
former smoker	446 (40.7)	86 (39.1)	92 (42.0)	91 (41.4)	76 (34.7)	77 (35.0)	96 (43.8)	92 (41.8)	101 (46.1)	85 (38.6)	105 (47.9)	72 (32.7)	89 (40.6)
current smoker	44 (4.0)	11 (5.0)	9 (4.1)	14 (6.4)	9 (4.1)	12 (5.5)	12 (5.5)	10 (4.5)	9 (4.1)	8 (3.6)	11 (5.0)	10 (4.5)	6 (2.7)
time since last meal, n (%)													
<2 h ago	634 (57.8)	129 (58.6)	136 (62.1)	128 (58.2)	127 (58.0)	117 (53.2)	135 (61.6)	129 (58.6)	125 (57.1)	132 (60.0)	129 (58.9)	132 (60.0)	131 (59.8)
2–4 h ago	411 (37.5)	82 (37.3)	73 (33.3)	82 (37.3)	81 (37.0)	92 (41.8)	76 (34.7)	84 (38.2)	78 (35.6)	81 (36.8)	77 (35.2)	76 (34.5)	84 (38.4)
>4 h ago	51 (4.7)	9 (4.1)	10 (4.6)	10 (4.5)	11 (5.0)	11 (5.0)	8 (3.7)	7 (3.2)	16 (7.3)	7 (3.2)	13 (5.9)	12 (5.5)	4 (1.8)
year of blood draw, *n* (%)													
1999	324 (29.6)	37 (16.8)	103 (47.0)	61 (27.7)	97 (44.3)	47 (21.4)	96 (43.8)	20 (9.1)	153 (69.9)	66 (30.0)	82 (37.4)	28 (12.7)	92 (42.0)
2000	711 (64.9)	165 (75.0)	114 (52.1)	151 (68.6)	120 (54.8)	162 (73.6)	113 (51.6)	183 (83.2)	47 (21.5)	130 (59.1)	135 (61.6)	177 (80.5)	124 (56.6)
2001	61 (5.6)	18 (8.2)	2 (0.9)	8 (3.6)	2 (0.9)	11 (5.0)	10 (4.6)	17 (7.7)	19 (8.7)	24 (10.9)	2 (0.9)	15 (6.8)	3 (1.4)

a Note: Q1, the first quintile, the lowest one-fifth of the exposure levels among the study population; Q5, the fifth quintile, the highest one-fifth of the exposure levels among the study population. PM_2.5_, fine particulate matter; PM_10_, coarse particulate matter; NO_2_, nitrogen dioxide; O_3_, ozone; SO_2_, sulfur dioxide; CO, carbon monoxide.

b American Cancer Society final dietary score. The diet score (ranges from 0–9) takes three aspects into account, including the servings of a variety of vegetables and fruits, the percentage of grains consumed as whole grains, and the servings of processed and red meats consumed. A higher diet score indicates that the individual takes a healthier diet.

**Table 2:** Air Pollutant Assessments of the Study Population (*N* = 1096)^a^

air pollutant assessments	overall (*N* = 1096) mean (SD)	Q1 median	Q5 median
PM_2.5_ (μg/m^3^)	12.821 (3.065)	9.498	15.794
PM_10_ (μg/m^3^)	21.558 (6.538)	14.978	28.382
NO_2_ (ppb)	13.822 (5.886)	7.496	20.617
O_3_ (ppb)	48.074 (6.984)	41.276	58.903
SO_2_ (ppb)	3.580 (1.761)	1.691	6.074
CO (ppm)	0.475 (0.179)	0.303	0.694

a Note: PM_2.5_, fine particulate matter; PM_10_, coarse particulate matter; NO_2_, nitrogen dioxide; O_3_, ozone; SO_2_, sulfur dioxide; CO, carbon monoxide. SD, standard deviation; Q1, the first quintile of exposure levels of study population; Q5, The fifth quintile of exposure levels of study population.

**Figure 1:**
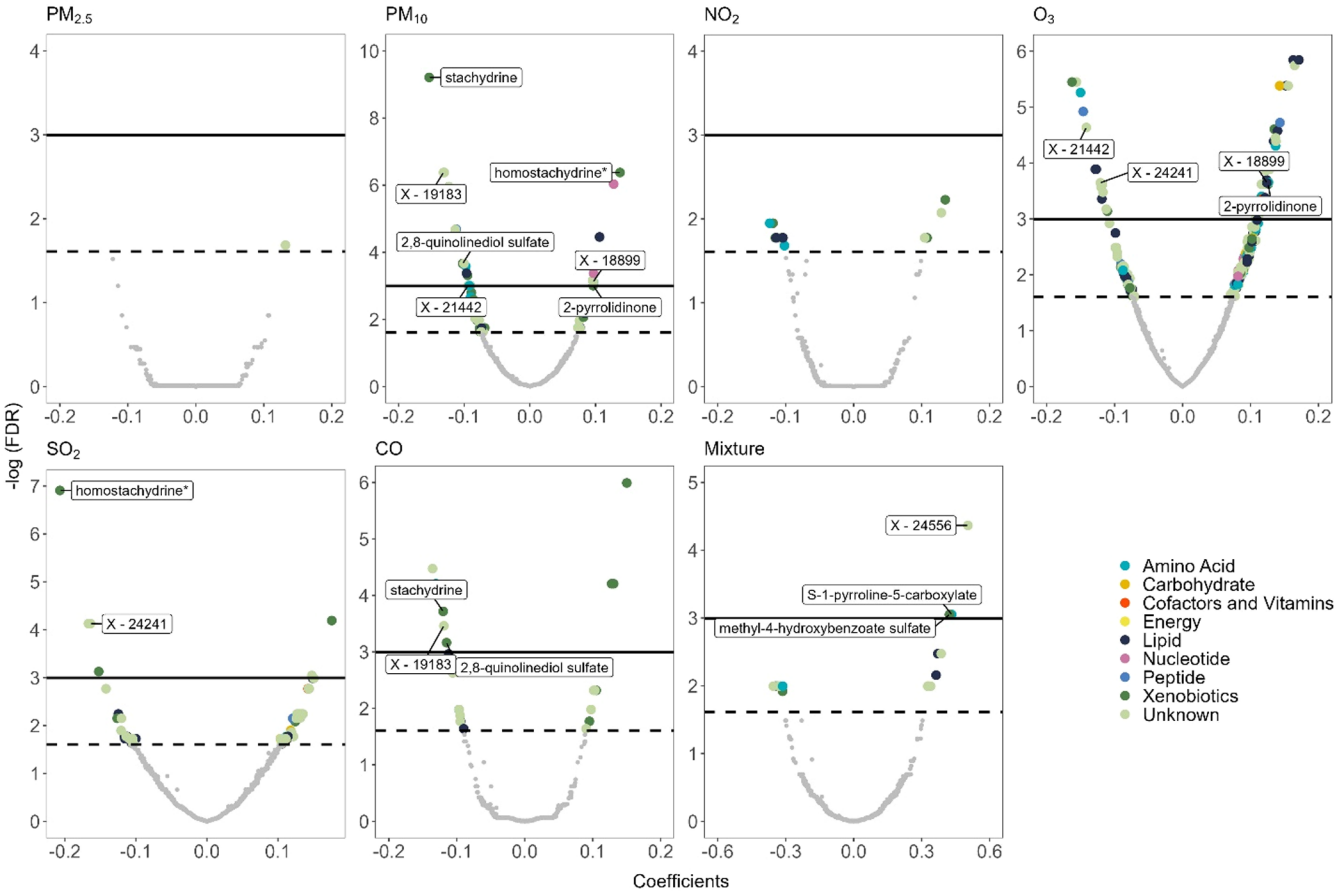
Volcano plots of associations between metabolite intensities and individual air pollutants or air pollution mixture. The *x*-axis denotes the coefficients of metabolite-pollutant associations. For individual air pollutant model, the coefficient is the change in natural log-transformed standardized metabolite intensity per interquartile range increase in air pollutant exposure levels. For the air pollution mixture model, the coefficient is the change in natural log-transformed standardized metabolite intensity per two quartiles (50%) increase in all air pollutant exposure levels. The *y*-axis denotes the negative natural log of false discovery rate (FDR) in metabolite-pollutant association. Different colors were used to represent different pathways where the metabolites are involved. The black solid line represents FDR = 0.05 and the black dashed line represents FDR = 0.2. For individual air pollutant model, the labeled metabolites were associated with two air pollutants. For the air pollution mixture model, the labeled metabolites were those meeting FDR < 0.05. PM_2.5_, fine particulate matter; PM_10_, coarse particulate matter; NO_2_, nitrogen dioxide; O_3_, ozone; SO_2_, sulfur dioxide; CO, carbon monoxide; Mixture, air pollution mixture. *A compound that has not been confirmed based on a standard, but Metabolon is confident in its identity (not tier 1).

For the individual air pollutant MWAS models, 92 unique metabolites (58 confirmed metabolites with known identities and 34 unknown) were significantly associated with at least one air pollutant (FDR < 0.05) (Table S1). We observed 31, 55, 6, and 8 metabolites significantly associated with PM_10_, O_3_, SO_2_, and CO, respectively (Figure 1 and Tables S1 and S2). We did not observe any metabolites significantly associated with PM_2.5_ and NO_2_ at FDR < 0.05. Eight metabolites were associated with two pollutants, four of which were xenobiotics and four unknown metabolites. Among them, three metabolites were significantly associated with both PM_10_ and O_3_ with consistent direction of effect, which were 2-pyrrolidinone and two unknown metabolites (X-18899 and X-21442). Additionally, three metabolites, including 2,8-quinolinediol sulfate, stachydrine, and an unknown metabolite X-19183, were significantly associated with both PM_10_ and CO exposure with a consistent direction of effect. One metabolite, homostachydrine, was positively associated with PM_10_ and negatively associated with SO_2_. One unknown metabolite, X-24241, is negatively associated with both O_3_ and SO_2_.

For the air pollution mixture MWAS models, we observed three metabolites significantly associated with the air pollution mixture (FDR < 0.05), which were S-1-pyrroline-5-carboxylate, methyl-4-hydroxybenzoate sulfate, and X-24556 (Figure 1 and Tables S1 and S2). The weights of each air pollutant to the overall effect estimate for these three metabolites are shown in Figure S5. According to quantile g-computation weights, SO_2_ and PM_10_ contributed the most to the overall mixture effect for S-1-pyrroline-5-and X-24556, while PM_10_ and CO contributed the most to the overall mixture effect for methyl-4-hydroxybenzoate sulfate. The number of significant metabolites identified in mixture models was much smaller than that identified in individual air pollutant models (Table S2). We did not observe any overlapping metabolites identified by both individual air pollutant models and mixture models at FDR < 0.05. However, several overlapping metabolites were identified by both individual air pollutant models and mixture models at FDR < 0.2 and unadjusted *P* < 0.05 (Figure S6).

All of the MWAS results are provided in the Supporting Information (Tables S1–S7).

Notably, when comparing to the known metabolites with level 1 or level 2 confidence reported in previous air pollution metabolomics studies,^47^ we were able to replicate 21 metabolites using both individual air pollutant models and air pollution mixture model, including taurine, creatinine, sebacate, oleoyl ethanolamide, palmitoyl ethanolamide (PEA), sphingomyelin (d18:2/18:1), and γ-glutamylvaline, and several others, in our study (Table S8).

**Figure 2:**
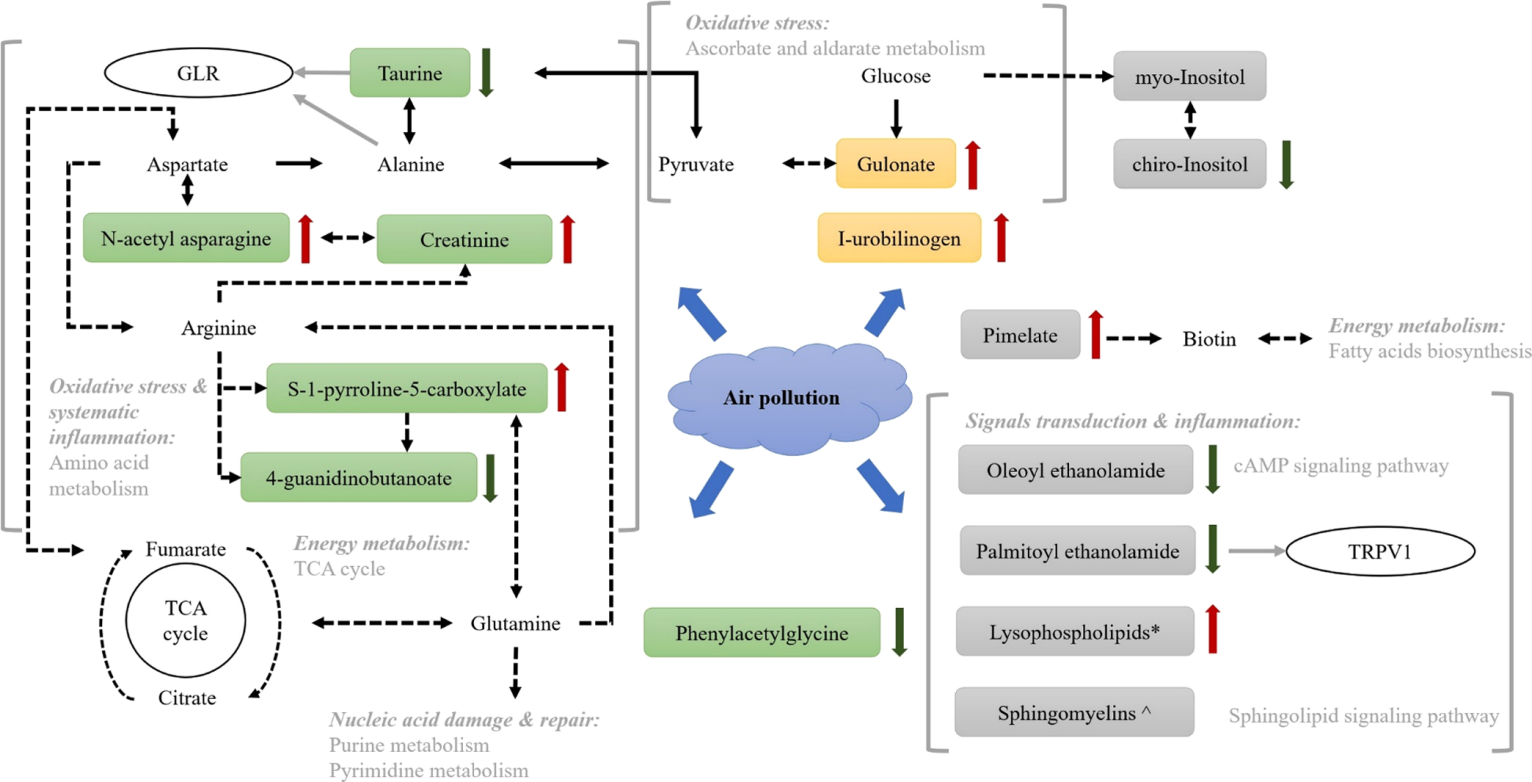
Potential molecular mechanism underlying the ambient air pollution toxicity using high-resolution metabolomics in the Cancer Prevention Study-II Nutrition cohort. Colored molecules are those identified in our study, with different colors corresponding to different biomolecule categories. Green molecules are amino acids/peptides; yellow molecules are cofactors/vitamins; gray molecules are lipids. The red arrow denotes the metabolite in positive association with air pollution exposure level, while the dark green arrow denotes the metabolite in negative association with air pollution exposure level. The solid black arrow indicates a single-step reaction between the molecule at the arrow’s end and the molecule at the arrow’s top, while the dashed black arrow indicates multiple steps between the molecule at the arrow’s end and the molecule at the arrow’s top. The gray arrow indicates the reaction between molecules and their corresponding receptors. TCA: citric acid cycle; GLR: glycine receptor α-1; TRPV1: transient receptor potential cation channel subfamily V member 1. *Three lysophospholipids were found positively associated with ozone, which were 1-linoleoyl-GPG (18:2), 1-oleoyl-GPE (18:1), and 2-stearoyl-GPE (18:0). ^∧^Two sphingomyelins were associated with air pollution: sphingomyelin (d18:2/18:1) was in positive association with coarse particulate matter, while sphingomyelin (d18:1/24:1, d18:2/24:0) was in negative association with ozone.

Considering both individual air pollutant models and air pollution mixture models, the greatest percentage of the 60 significant confirmed metabolites with known identities was those involved in xenobiotic metabolism-related pathways (33%). The remaining confirmed metabolites were those enriched in lipids (28%) and amino acids (22%) metabolic pathways (Table S1 and Figure 1). Further, the known metabolites were closely linked to pathways involved in oxidative stress and systemic inflammation (e.g., urea cycle; arginine and proline metabolism; tryptophan metabolism; leucine, isoleucine, and valine metabolism, ascorbate, and aldarate metabolism), energy metabolism (e.g., fatty acid metabolism, TCA cycle), signals transduction (e.g., sphingolipid metabolism, lysophospholipid, endocannabinoid), nucleic acid damage and repair (i.e., purine and pyrimidine metabolism), and xenobiotic pathways, which collectively reveal the potential molecular mechanisms underlying air pollution toxicity on human metabolome (Figure 2).

To test the robustness of our results with respect to adjustments for potential confounding and temporal misalignment, we conducted several sensitivity analyses. In general, we observed consistent findings with robust effect estimates across different sets of sensitivity analyses. For individual air pollutants models, after including breast cancer status in the models, the number and identities of most air pollutants associated features remained the same, except for three metabolites that were previously significantly associated with O_3_ became insignificant (Table S9). Additionally, the effect coefficients remained robust and consistent (Figure S7). When using the annual average exposures in the previous year of blood draw as exposure indicators (*N* = 772), the number of significant metabolites reduced from 92 to 31, with 20 overlapping metabolites consistently identified (Figure S8 and Table S10). As for the separate analyses among never-smokers (*N* = 606) and controls (*N* = 528), the effect estimates from linear models were similar and correlated with those from the main analyses (Figures S9 and S10). The number of significant features among never-smokers decreased from 92 to 76, with 41 overlapping metabolites (Table S11). Only one metabolite, 1-linoleoyl-GPG (18:2), remained significantly associated with the occurrence of O_3_ among controls (Table S12). For air pollution mixture models, three additional significant metabolites were identified after further including breast cancer status, which were taurine, sphingadienine, and choline (Table S13). We did not find any significant metabolites among never-smokers, controls, and when using the annual average exposures in the previous year of blood draw as exposure indicators, which was possibly due to the smaller sample size resulting in less statistical power. The effect coefficients remained robust and consistent (Figure S11).

## Discussion

In this large-scale cross-sectional metabolome-wide association study among 1096 women within the well-established CPS-II Nutrition Cohort, we evaluated the effects of individual air pollutants and overall air pollution mixture on serum metabolome. Our study findings added additional novel insights to the very limited number of existing air pollution mixture analyses investigating the potential joint effects of air pollution on human metabolome.^48,49^ We detected numerous metabolites significantly associated with long-term exposure to air pollution and verified metabolites that were closely linked and connected in key inflammatory, redox, energy metabolism, signal transduction, and nucleic acid damage and repair pathways. Among the current results, we successfully replicated 21 metabolites previously reported in other independent panel and cohort studies,^20−34^ and identified an additional 39 novel metabolites that have not been reported in air pollution studies previously, all of which have been confirmed with level 1 or 2 evidence and may potentially be further developed as sensitive biomarkers for assessing internal exposures to air pollution. Collectively, these findings point to several potential molecular mechanisms of ambient air pollution toxicity.

Our group recently published a state-of-the-science review on high-resolution metabolomics applications in air pollution health research,^42^ which summarizes current progress, analytical challenges, and directions for future research. The review included 47 articles published between January 1, 2005, and March 31, 2022. By comparing our findings with previously reported known metabolites with level 1 or level 2 confidence in air pollution metabolomics studies, we replicated 21 metabolites, including taurine, creatine, and sebacate.^42^ A detailed list of metabolites associated with various air pollutants can be found in the Supporting Information of Liang et al.^42^ Many of the identified air pollution-associated metabolites in our study were involved in biological pathways, including the urea cycle; alanine and aspartate metabolism, tryptophan metabolism, leucine, isoleucine, and valine metabolism, fatty acid metabolism, TCA cycle, purine and pyrimidine metabolism, and sphingolipid metabolism, which were closely linked to oxidative stress, inflammatory responses, energy metabolism, signal transduction, and nucleic acid damage effect. Importantly, these specific pollution-mediated pathways have also been reported in other panel studies and cohorts to be associated with various air pollution components and adverse health effects, including respiratory and cardiovascular diseases, and adverse reproductive and birth outcomes.^22−24,27,28,42,50,51^ Nevertheless, it is worth noting that for a certain number of metabolite-air pollutant associations, discrepancies exist in the association direction between the present study and previous studies. Several important factors may contribute to the discrepancies across studies.^42^ First, the type of biospecimen used for metabolomics profiling, such as serum, plasma, urine, and exhaled breath, can impact the detection of metabolic changes associated with air pollution exposures as different biospecimens may reflect distinct metabolic processes. Second, differences in the exposure time window, such as short-term versus long-term exposures, could reveal different metabolic responses because the half-life of metabolites varies widely, with some changes detectable only immediately after exposure and others persisting over a long period. Third, variations in the protocols, including analytic platforms, chemical annotation, and confirmation, could significantly affect the detected metabolic changes linked to air pollution exposure. Fourth, our study population was mostly elderly white, limiting the generalizability and potentially contributing to differences in metabolic responses compared with more diverse populations, as age and race can significantly influence both baseline metabolomics profiles and responses to air pollution exposures. Notably, we identified 39 novel metabolites associated with chronic air pollution exposure that have not been reported before. However, the documentation of functions of these metabolites were limited. Future studies should validate our results and further explore the roles of these metabolites.

Of particular note were pathways associated with lipid metabolism, including fatty acid, lysophospholipid, endocannabinoid, and sphingolipid metabolism. Fatty acid metabolism has previously been associated with near-roadway air pollution, NO_2_, O_3_, and PM exposure,^52,53^ specifically monohydroxy fatty acid metabolism.^53^ Three lysophospholipids, also known as lysolipids, were positively associated with exposure to O_3_ in our analysis. This is consistent with a previous study that also found that lysophospholipids were elevated in serum samples following O_3_ exposure.^53^ These lysophospholipids interact with lysophospholipid membrane receptors, impacting inflammation and energy production.^53,54^ Two endocannabinoids, oleoyl ethanolamide and palmitoyl ethanolamide, were negatively associated with exposure to O_3_. Endocannabinoids have been found to have anti-inflammatory and immune-suppressive properties and act as neuronal protection. Specifically, metabolomic changes in palmitoyl ethanolamide was positively associated with exposure to mixed gasoline and diesel emissions.^55^ Another study demonstrated the use of endocannabinoids as protection against neuroinflammation relating to SO_2_ exposure.^56^ Additionally, endocannabinoid synthesis had a significant fold enrichment value for O_3_ exposure.^53^ The decreased intensities of the endocannabinoid metabolites in relation to the increased air pollutant exposure level may demonstrate a protective role in reducing inflammation. Consistently in our study, we also observed a positive association between PM_10_ and a negative association between O_3_ and metabolites involved with sphingolipid metabolism. Sphingolipids are a class of lipids primarily functioning as structural molecules within cellular membranes and regulators of biological processes within cancer cell signal transduction.^57^ Previous studies have also found associations between perturbations in sphingolipid metabolism and NO_2_, O_3_, and PM_2.5_ exposure.^53,58−60^

Our results also revealed several metabolites involved in oxidative stress and systemic inflammation-related pathways that were associated with ambient air pollution exposures. Oxidative stress is caused by chemical imbalances between oxidative and antioxidative systems in the body, which may cause an excess production of free radicals, such as reactive oxygen or nitrogen species.^61^ Many amino acids act as modifiers for reactive species, leading to oxidative stress in the body. In our study, we identified multiple pathways of amino acid metabolism that were significantly associated with exposure to PM_10_ and O_3_, including the urea cycle, tryptophan metabolism, methionine, cysteine, SAM, and taurine metabolism. Metabolites involved in tryptophan metabolism were positively associated with PM_10_ exposure and negatively associated with CO exposure. Tryptophan is an amino acid metabolized through kynurenine and serotonin pathways, contributing to various pathophysiological pathways, including inflammation, immune responses, and neurological function.^62,63^ Previous studies have associated exposure to traffic-related air pollution with tryptophan metabolism, specifically in-vehicle particulate metals, which make up PM_10_ and PM_2.5_ mixtures, as well as O_3_ exposure.^22,64,65^ We also observed a negative association between taurine and exposure to O_3_. Taurine is an antioxidant that can help scavenge reactive oxygen species.^66,67^ However, the results are not consistent across the existing air pollution metabolomics studies. A study of healthy adults found that short-term O_3_ was positively associated with taurine levels in bronchioalveolar lavage fluid.^65^

Energy disruption and nucleic acid damage-related pathways and metabolites were found to be associated with chronic exposure to ambient air pollution in our study. Specifically, we observed a positive association between a metabolite in the TCA cycle with O_3_. TCA cycle is the major energy-producing metabolic pathway in cells, by oxidating acetyl-coenzyme A derived from carbohydrates, proteins, and fatty acids.^68^ In cell stress conditions, TCA cycle intermediates may be released from the mitochondrial membrane into the cytosol due to the disruption of the mitochondrial membrane, which has an impact on the cellular immunity.^68^ In addition, we observed changes in metabolites in purine and pyrimidine metabolism in association with long-term exposure to PM_10_. Purine and pyrimidine metabolism are essential mechanisms in DNA damage and repair pathways and have also been associated with multiple air pollutants including PM and PM components in other studies.^24,50,69^

Additionally, we observed a substantial proportion of air pollution-associated metabolites in xenobiotic pathways, encompassing four chemical metabolites and 12 metabolites derived from food components/plants, which may suggest potential coexposures to other pollutants or the influence of residual confounding. For instance, 4-hydroxychlorothalonil found in our study is a metabolite of chlorothalonil, a widely used fungicide on both crop protection and wood preservatives.^70^ Several food-related metabolites (e.g., alliin, erythritol, and theanine) implicated the potential confounding effects of dietary factors, despite our control of a diet score and time since the last meal in the main analysis. Thus, our results should be interpreted with caution and future studies should consider investigating the coexposures of environmental pollutants and collecting fasting blood samples for metabolic profiling.

We did not observe any associations with PM_2.5_ but did identify PM_10_-associated metabolites at FDR < 0.05. This finding contrasts with extensive evidence of PM_2.5_ health impacts. While we could not identify the real cause for this observation, several factors may account for this null finding. First, our analysis relied on annual average PM_2.5_ mass exposure levels, which do not account for the variation in the chemical composition and corresponding carcinogenic potential across different locations. A recent study has reported the differences in contributions to the oxidative potential of different components in particles.^71^ The participants in our study were from diverse states across the U.S., likely experiencing heterogeneous PM compositions, which may lead to variations in toxicity. Future PM_2.5_ metabolomics studies will likely benefit from validated prediction models for PM components. Second, the prediction model used for PM_10_ is less accurate than that for PM_2.5_, potentially introducing uncertainty in exposure estimates.^40^ Third, the relatively low concentration and limited variability of the PM_2.5_ exposure levels among participants may not have been sufficient to detect any metabolic changes associated with PM_2.5_.

In general, we identified fewer significant metabolites associated with air pollution mixtures than individual air pollutants, contrary to our expectation. Building on previous evidence, we hypothesized potential additive or synergic effects among air pollutants,^72^ which might enable us to detect more extensive metabolic perturbation. While there is no clear explanation for this observation, one potential cause may be that using prediction models with varying performance for exposure assessment on different air pollutants may lead to uncertainties in the exposure characterization, which could, in turn, introduce and amplify uncertainties when assessing the joint effects of all air pollutants. Moreover, the findings revealed limited consistency between individual air pollutant models and air pollution mixture models using quantile g-computation, as demonstrated by no overlapping significant metabolites at FDR < 0.05, although some overlap was observed at looser thresholds of FDR < 0.2 and unadjusted *P* < 0.05. Future large-scale metabolomics studies are warranted to continue examining air pollution as a mixture to better assess the potential joint effects on human metabolome.

This study has several strengths, including a large sample size, consideration of the overall air pollution mixture effect, use of a well-established cohort with documented long-term air pollution-related mortality, covariate control, stringent false discovery rate correction to adjust for multiple testing, and advanced metabolic profiling and chemical annotation with over 70% of metabolites confirmed with level 1 or 2 evidence. Despite this, certain limitations deserve specific attention. First, the cross-sectional study design reduced our ability to explore temporal variation of air pollution and trajectories in metabolomic perturbations, which is an important determinant in establishing causal inference. Additionally, temporal misalignment was possible, meaning that part of the exposure measurement period occurs after biosample collection. Future studies should consider repeated measurements to comprehensively characterize longitudinal metabolomics responses to air pollution exposure. Second, although we used validated spatiotemporal models to estimate air pollution exposure at participants’ residential addresses, we lacked data on their time-activity patterns and indoor exposures (e.g., cooking activities). Consequently, this is still an imperfect proxy of personal exposure, which would likely result in nondifferential exposure misclassification.^73,74^ Third, nonfasting blood samples may introduce variation in the metabolomics profiles given the potential introduction of diet-related metabolites. Yet, to mitigate this effect, we included hours since the last meal as a covariate in our analyses, as shown in various air pollution metabolomics investigations. Additionally, we utilized pool standards and internal references in the metabolic profiling, and followed a thorough metabolomics workflow to mitigate the potential effects of nonfasting status, as successfully demonstrated in previous studies.^75−77^ Fourth, among the numerous metabolites that we identified, there is a substantial risk of false positives due to multiple comparisons and Type 1 errors. To minimize this, we applied a stringent significance cutoff at 0.05 after the multiple comparison correction, and several significant metabolites identified in our study have been consistently reported in previous studies. Finally, this study was conducted among older females that were mostly white and generally of a higher socioeconomic status. Caution is warranted when extrapolating the findings to other more diverse populations.

## Supporting Information Available

Table S1 for metabolites significantly associated with air pollution (FDR < 0.05). Table S2 for the number of metabolites associated with individual air pollutant or air pollution mixture exposure levels at different thresholds for the main analysis and sensitive analyses. Figure S1 for flowchart of exclusion criteria for study participants in the final analysis. Figure S2 for a directed acyclic graph of relationships among air pollution exposure, metabolite intensity, and covariates. Figure S3 for distribution of residential states for study population. Figure S4 for a heatmap of Pearson correlations among average exposure levels in the year of blood draw of six air pollutants. Figure S5 for weights of individual air pollutants for significant metabolites in air pollution mixture-metabolites models (FDR < 0.05). Figure S6 for overlapping metabolites associated with individual air pollutant exposure and air pollution mixture at false discovery rate (FDR) < 0.2 and unadjusted *P* < 0.05. Figure S7 for relations of effect coefficients of metabolite-pollutants associations from metabolome-wide association study (MWAS) models before and after adjusting for breast cancer status. Figure S8 for relations of effect coefficients of metabolite-pollutants associations from metabolome-wide association study (MWAS) models between using annual average exposure in the current year of the blood draw and using annual average exposure in the previous year of the blood draw as exposure indicator. Figure S9 for relations of effect coefficients of metabolite-pollutants associations from metabolome-wide association study (MWAS) between the overall study population and among the never-smoker group. Figure S10 for relations of effect coefficients of metabolite-pollutants associations from metabolome-wide association study (MWAS) between the overall study population and among the control group. Figure S11 for relations of effect coefficients from air pollution mixture models between main analysis and sensitivity analyses (PDF)
Table S1 for metabolome-wide association study results for all metabolite-fine particulate matter models. Table S2 for metabolome-wide association study results for all metabolite-coarse particulate matter models. Table S3 for metabolome-wide association study results for all metabolite-nitrogen dioxide models. Table S4 for metabolome-wide association study results for all metabolite-ozone models. Table S5 for metabolome-wide association study results for all metabolite-sulfur dioxide models. Table S6 for metabolome-wide association study results for all metabolite-carbon monoxide models. Table S7 for metabolome-wide association study results for air pollution mixture models. Table S8 for air pollution-associated metabolites identified in the present study that have also been reported in other previous studies. Table S9 for significant metabolites associated with air pollutants for models further adjusting for breast cancer status. Table S10 for significant metabolites associated with air pollutants using annual average exposure in the previous year of the blood draw as exposure indicator. Table S11 for significant metabolites associated with air pollutants in the never-smoker group. Table S12 for significant metabolites associated with air pollutants in the control group. Table S13 for significant metabolites associated with air pollution mixture models further adjusting for breast cancer status. (XLSX)
