# Supplementary material for: Metabolomics Signatures of Exposure to Ambient Air Pollution: A Large-Scale Metabolome-Wide Association Study in the Cancer Prevention Study-II Nutrition Cohort
Source: Environ Sci Technol. 2024 Dec 16;59(1):212–23. doi: 10.1021/acs.est.4c09592 (PMC11741098; doi:10.1021/acs.est.4c09592)
Supplement: Supplementary file 1 — es4c09592_si_001.pdf [file es4c09592_si_001.pdf]

Supporting Information

Metabolomics Signatures of Exposure to Ambient Air Pollution: A Large-Scale Metabolome-Wide Association Study in the Cancer Prevention Study-II Nutrition Cohort

Donghai Liang[1*$], Ziyin Tang[1$], W.Ryan Diver[2,4,5], Jeremy A. Sarnat[1], Sabrina S. Chow[1], Haoran Cheng[1], Emily L. Deubler[2], Youran Tan[1], Stephanie M. Eick[1], Michael Jerrett[3], Michelle C. Turner[4-6], Ying Wang[2*]

[1] Gangarosa Department of Environmental Health, Rollins School of Public Health, Emory University, Atlanta, Georgia, United States, 30322

[2] Department of Population Science, American Cancer Society, Atlanta, Georgia, United States, 30303

[3] Department of Environmental Health Sciences, Fielding School of Public Health, University of California Los Angeles, Los Angeles, California, United States, 90095

[4] Barcelona Institute for Global Health (ISGlobal), Barcelona, Spain, 08036

[5] Universitat Pompeu Fabra (UPF), Barcelona, Spain, 08018

[6] CIBER Epidemiología y Salud Pública (CIBERESP), Madrid, Spain, 28029

$D.L. and Z.T. contributed equally to this paper.

***Corresponding Authors**

Donghai Liang, PhD

Assistant Professor, Gangarosa Department of Environmental Health

Emory University Rollins School of Public Health

1518 Clifton Rd

Atlanta, GA 30322, USA

donghai.liang@emory.edu

Ying Wang, PhD

Senior Principal Scientist, Epidemiology Research

American Cancer Society, Inc.

270 Peachtree Street NW Suite 1300

Atlanta, GA 30303

ying.wang@cancer.org

Conflict of Interest Statement: *The authors declare they have nothing to disclose.*

Number of pages	18
Number of figures	11
Number of tables	2

Table S1. Metabolites significantly associated with air pollution (FDR < 0.05) [a].

Metabolite	Mass	RI	Platform	Pathway	Sub pathway	Percent change [b]	FDR
**PM_{10}**							
4-guanidinobutanoate	146.0924	2320.0	LC/MS Pos Early	Amino Acid	Guanidino and Acetamido Metabolism	-9.3%	0.0278
N-trimethyl 5-aminovalerate	160.1332	2186.0	LC/MS Pos Early	Amino Acid	Lysine Metabolism	-8.8%	0.0495
tryptophan betaine	247.1441	2673.0	LC/MS Pos Early	Amino Acid	Tryptophan Metabolism	10.2%	0.0442
N-methylproline	130.0863	1335.0	LC/MS Pos Early	Amino Acid	Urea cycle; Arginine and Proline Metabolism	-11.6%	0.0026
eicosapentaenoylcholine	388.321	1343.0	LC/MS Pos Late	Lipid	Fatty Acid Metabolism (Acyl Choline)	-9.2%	0.0342
3-carboxy-4-methyl-5-propyl-2-furanpropanoate (CMPF)	239.0925	2840.0	LC/MS Neg	Lipid	Fatty Acid, Dicarboxylate	-9.7%	0.0256
chiro-inositol	225.0616	3191.2	LC/MS Polar	Lipid	Inositol Metabolism	-11.6%	0.0026
sphingomyelin (d18:2/18:1)*	727.5749	1904.0	LC/MS Pos Late	Lipid	Sphingolipid Metabolism	11.2%	0.0116
N1-methylinosine	283.1037	1430.0	LC/MS Pos Early	Nucleotide	Purine Metabolism, (Hypo)Xanthine/Inosine containing	10.2%	0.0342
2'-deoxyuridine	227.0673	1586.0	LC/MS Neg	Nucleotide	Pyrimidine Metabolism, Uracil containing	13.6%	0.0024
phenylacetylglycine	192.0666	2375.0	LC/MS Neg	Peptide	Acetylated Peptides	-10.6%	0.0092
gamma-glutamylvaline	247.1289	2700.0	LC/MS Pos Early	Peptide	Gamma-glutamyl Amino Acid	-8.7%	0.0495
4-ethylphenylsulfate	201.0227	3580.0	LC/MS Neg	Xenobiotics	Benzoate Metabolism	-10.7%	0.0095
2-pyrrolidinone [c]	86.06	1262.0	LC/MS Pos Early	Xenobiotics	Chemical	10.1%	0.0495
2,8-quinolinediol sulfate [d]	239.9972	2612.0	LC/MS Neg	Xenobiotics	Food Component/Plant	-9.7%	0.0256
homostachydrine* [e]	158.1176	1750.0	LC/MS Pos Early	Xenobiotics	Food Component/Plant	14.7%	0.0017
methyl glucopyranoside (alpha + beta)	193.0718	1070.0	LC/MS Neg	Xenobiotics	Food Component/Plant	-9.1%	0.0364
stachydrine [d]	144.1019	1440.0	LC/MS Pos Early	Xenobiotics	Food Component/Plant	-14.2%	0.0001
X - 02269	255.0876	1525.4	LC/MS Neg	Unknown		-11.1%	0.0032
X - 16947	345.1559	3802.0	LC/MS Neg	Unknown		-12.2%	0.0017
X - 17145	257.1761	3862.0	LC/MS Neg	Unknown		-8.7%	0.0495
X - 17350	345.1564	2721.0	LC/MS Neg	Unknown		-11.3%	0.0034
X - 17654	365.2694	5200.0	LC/MS Neg	Unknown		-10.6%	0.0123
X - 18899 [c]	337.1427	3730.0	LC/MS Neg	Unknown		10.1%	0.0442
X - 19183 [d]	230.0495	1665.0	LC/MS Neg	Unknown		-12.3%	0.0017
X - 21442 [c]	333.2077	3823.0	LC/MS Neg	Unknown		-9.0%	0.0495
X - 21844	220.0987	4010.0	LC/MS Neg	Unknown		10.0%	0.0423
X - 22515	345.1563	3869.0	LC/MS Neg	Unknown		-11.4%	0.0029

X - 22836	146.0812	1200.0	LC/MS Pos Early	Unknown		-10.7%	0.0093
X - 23314	207.0872	1578.3	LC/MS Neg	Unknown		-9.5%	0.0256
X - 24738	160.0967	1051.0	LC/MS Pos Early	Unknown		-11.6%	0.0026
**O_3**							
N-acetylasparagine	175.0713	785.0	LC/MS Pos Early	Amino Acid	Alanine and Aspartate Metabolism	14.7%	0.0135
creatinine	114.0662	2055.0	LC/MS Pos Early	Amino Acid	Creatine Metabolism	13.5%	0.0259
N-acetyl-1-methylhistidine*	212.1028	2100.0	LC/MS Pos Early	Amino Acid	Histidine Metabolism	13.3%	0.0259
N-acetylvaline	158.0823	1704.0	LC/MS Neg	Amino Acid	Leucine, Isoleucine and Valine Metabolism	12.3%	0.0333
isovalerylglycine	158.0823	1950.0	LC/MS Neg	Amino Acid	Leucine, Isoleucine and Valine Metabolism	13.4%	0.0205
taurine	124.0074	690.0	LC/MS Neg	Amino Acid	Methionine, Cysteine, SAM and Taurine Metabolism	-13.9%	0.0052
N-alpha-acetylornithine	175.1077	2000.0	LC/MS Pos Early	Amino Acid	Urea cycle; Arginine and Proline Metabolism	12.2%	0.0399
erythronate*	135.0299	2186.0	LC/MS Polar	Carbohydrate	Aminosugar Metabolism	15.3%	0.0046
gulonate*	195.051	2750.0	LC/MS Polar	Cofactors and Vitamins	Ascorbate and Aldarate Metabolism	12.2%	0.0408
I-urobilinogen	591.3188	4190.0	LC/MS Neg	Cofactors and Vitamins	Hemoglobin and Porphyrin Metabolism	12.4%	0.0348
succinylcarnitine (C4-DC)	262.1285	2291.0	LC/MS Pos Early	Energy	TCA Cycle	11.8%	0.0496
oleoyl ethanolamide	324.2908	6450.0	LC/MS Neg	Lipid	Endocannabinoid	-11.9%	0.0205
palmitoyl ethanolamide	298.2752	6300.0	LC/MS Neg	Lipid	Endocannabinoid	-12.0%	0.0205
pimelate (heptanedioate)	159.0663	2745.0	LC/MS Polar	Lipid	Fatty Acid, Dicarboxylate	14.4%	0.0124
sebacate (decanedioate)	201.1132	1788.0	LC/MS Neg	Lipid	Fatty Acid, Dicarboxylate	13.2%	0.0248
suberate (octanedioate)	173.0819	2577.6	LC/MS Polar	Lipid	Fatty Acid, Dicarboxylate	16.5%	0.0046
5-hydroxydecanoate	187.134	4080.0	LC/MS Neg	Lipid	Fatty Acid, Monohydroxy	17.7%	0.0029
5-hydroxyhexanoate	131.0714	1267.0	LC/MS Neg	Lipid	Fatty Acid, Monohydroxy	12.3%	0.0367
1-linoleoyl-GPG (18:2)*	507.2728	5516.0	LC/MS Neg	Lipid	Lysophospholipid	18.7%	0.0029
1-oleoyl-GPE (18:1)	480.3085	1554.0	LC/MS Pos Late	Lipid	Lysophospholipid	12.9%	0.0341
2-stearoyl-GPE (18:0)*	480.3096	6350.0	LC/MS Neg	Lipid	Lysophospholipid	15.0%	0.0103
3-hydroxy-3-methylglutarate	161.0456	2700.0	LC/MS Polar	Lipid	Mevalonate Metabolism	13.2%	0.0261
glycoursodeoxycholate	448.3068	5033.0	LC/MS Neg	Lipid	Secondary Bile Acid Metabolism	12.6%	0.0367
sphingomyelin (d18:1/24:1, d18:2/24:0)*	813.6844	3033.0	LC/MS Pos Late	Lipid	Sphingolipid Metabolism	-11.2%	0.0348
4-hydroxyphenylacetylglutamine	279.0986	1650.0	LC/MS Neg	Peptide	Acetylated Peptides	15.4%	0.0089
phenylalanylglycine	223.1077	2898.0	LC/MS Pos Early	Peptide	Dipeptide	-13.6%	0.0073
4-hydroxyhippurate	194.0459	1475.0	LC/MS Neg	Xenobiotics	Benzoate Metabolism	14.8%	0.0103

2-pyrrolidinone	86.06	1262.0	LC/MS Pos Early	Xenobiotics	Chemical	13.4%	0.0268
3-hydroxypyridine sulfate	173.9867	1595.5	LC/MS Neg	Xenobiotics	Chemical	-11.1%	0.0268
4-hydroxychlorothalonil	244.9082	4427.0	LC/MS Neg	Xenobiotics	Chemical	-15.0%	0.0043
lanthionine	209.0591	1730.0	LC/MS Pos Early	Xenobiotics	Chemical	12.0%	0.0445
2,3-dihydroxypyridine	112.0393	1420.0	LC/MS Pos Early	Xenobiotics	Food Component/Plant	-10.5%	0.0434
2-piperidinone [c]	100.0757	1675.0	LC/MS Pos Early	Xenobiotics	Food Component/Plant	12.9%	0.0367
erythritol	167.0561	1491.0	LC/MS Polar	Xenobiotics	Food Component/Plant	14.5%	0.01
eugenol sulfate	243.0333	3782.0	LC/MS Neg	Xenobiotics	Food Component/Plant	12.2%	0.0431
X - 11334	259.0921	2017.0	LC/MS Pos Early	Unknown		13.7%	0.0205
X - 12462	148.0427	1790.0	LC/MS Pos Early	Unknown		16.8%	0.0046
X - 12816	495.2237	2700.0	LC/MS Neg	Unknown		-14.5%	0.0043
X - 13529	188.0928	2011.0	LC/MS Neg	Unknown		13.1%	0.021
X - 14473	211.1436	2553.0	LC/MS Pos Early	Unknown		-12.5%	0.014
X - 16570	198.1143	3765.0	LC/MS Neg	Unknown		14.7%	0.0124
X - 17179	595.7533	2480.0	LC/MS Neg	Unknown		-11.3%	0.0282
X - 18889	202.1087	2633.0	LC/MS Neg	Unknown		11.3%	0.0496
X - 18899 [c]	337.1427	3730.0	LC/MS Neg	Unknown		13.2%	0.0261
X - 21442 [c]	333.2077	3823.0	LC/MS Neg	Unknown		-13.2%	0.0097
X - 21467	262.6205	4041.0	LC/MS Neg	Unknown		14.7%	0.0117
X - 22771	160.0441	2180.0	LC/MS Neg	Unknown		17.9%	0.0032
X - 23583	116.0707	1112.0	LC/MS Pos Early	Unknown		13.5%	0.0259
X - 23649	272.076	1645.0	LC/MS Pos Early	Unknown		-11.1%	0.0309
X - 23655	110.0602	2200.0	LC/MS Pos Early	Unknown		-10.6%	0.0418
X - 23922	171.0663	1582.3	LC/MS Neg	Unknown		11.9%	0.0471
X - 24241 [f]	186.1124	2239.0	LC/MS Pos Early	Unknown		-11.4%	0.0261
X - 24337	239.0786	1980.6	LC/MS Neg	Unknown		12.3%	0.0268
X - 24494	479.2277	4131.0	LC/MS Neg	Unknown		12.8%	0.0268
X - 24811	228.0865	2259.5	LC/MS Pos Early	Unknown		-11.1%	0.0269
**SO_2**							
o-cresol sulfate	187.0071	2796.0	LC/MS Neg	Xenobiotics	Benzoate Metabolism	-14.1%	0.0438

homostachydrine*[e]	158.1176	1750.0	LC/MS Pos Early	Xenobiotics	Food Component/Plant	-18.7%	0.001
theanine	175.1077	2180.0	LC/MS Pos Early	Xenobiotics	Food Component/Plant	19.2%	0.0151
X - 11381	186.1123	2331.0	LC/MS Pos Early	Unknown		-15.4%	0.0161
X - 12906	158.0462	793.0	LC/MS Neg	Unknown		15.9%	0.0475
X - 24241[f]	186.1124	2239.0	LC/MS Pos Early	Unknown		-15.1%	0.0161
**CO**							
indole-3-carboxylic acid	160.0404	1708.8	LC/MS Neg	Amino Acid	Tryptophan Metabolism	-12.3%	0.0149
2,8-quinolinediol sulfate[d]	239.9972	2612.0	LC/MS Neg	Xenobiotics	Food Component/Plant	-10.9%	0.0423
N-acetylalliin	218.0493	1467.0	LC/MS Neg	Xenobiotics	Food Component/Plant	13.6%	0.0149
S-allylcysteine	162.0583	2690.0	LC/MS Pos Early	Xenobiotics	Food Component/Plant	14.0%	0.0149
alliin	178.0532	1470.0	LC/MS Pos Early	Xenobiotics	Food Component/Plant	16.2%	0.0025
stachydrine[d]	144.1019	1440.0	LC/MS Pos Early	Xenobiotics	Food Component/Plant	-11.3%	0.0243
X - 12798	240.1017	2011.0	LC/MS Pos Early	Unknown		-12.7%	0.0114
X - 19183[d]	230.0495	1665.0	LC/MS Neg	Unknown		-11.2%	0.0314
**Mixture**							
S-1-pyrroline-5-carboxylate	114.055	1528.0	LC/MS Pos Early	Amino Acid	Glutamate Metabolism	54.1%	0.0473
methyl-4-hydroxybenzoate sulfate	230.9969	2873.0	LC/MS Neg	Xenobiotics	Benzoate Metabolism	52.6%	0.0473
X - 24556	283.1191	2318.0	LC/MS Neg	Unknown		65.6%	0.0127

Note: FDR, false discovery rate; RI, retention index; PM_{10}, coarse particulate matter; O_3, ozone; SO_2, sulfur dioxide; CO, carbon monoxide; LC/MS, liquid chromatography/ mass spectrometry.

[a] Multiple linear regression models were used to examine the associations between iindividual air pollutants exposure levels and individual metabolites, while quantile g-computation models were used to examine the associations between overall air pollution mixture and individual metabolites. Both two approaches controlled for age at blood draw (continuous), body mass index (BMI; continuous), dietary score (continuous), race (categorical: white and non-white), smoking status (categorical: never, former, and current smoker), year of blood draw (categorical: 1999, 2000, and 2001), and hours since last meal (categorical: <2 hours ago, 2-4 hours ago, and >4 hours ago). Banjamini-Hochberg procedure was used to correct for multiple testing. [b] For individual air pollutant models, the effect estimates were expressed as the percent change in standardized metabolite intensity per interquartile range increase in air pollutant exposure levels. For the air pollution mixture models, the effect estimates were expressed as the percent change in standardized metabolite intensity per two quartiles (50%) increase in all air pollutant exposure levels. [c] Metabolites associated with both PM_{10} and O_3 exposure. [d] Metabolites associated with both PM_{10} and CO exposure. [e] Metabolites associated with both PM_{10} and SO_2 exposure. [f] Metabolites associated with both O_3 and SO_2 exposure. * A compound that has not been confirmed based on a standard, but Metabolon is confident in its identity (not tier 1).

Table S2. The number of metabolites associated with individual air pollutant or air pollution mixture exposure levels at different thresholds for the main analysis and sensitive analyses. The total number of metabolites included in the analyses was 1,186.

	P<0.05	*P*<0.005	FDR<0.2	FDR<0.05
**Main analysis (*N* = 1,096)**				
$PM_{2.5}$	63	9	1	0
PM_{10}	152	45	63	31
NO_2	82	23	18	0
O_3	264	78	246	55
SO_2	174	31	68	6
CO	81	27	27	8
Mixture	103	27	27	3
**Main analysis further adjusted for breast cancer status (*N* = 1,096)**				
$PM_{2.5}$	61	9	0	0
PM_{10}	149	45	60	31
NO_2	82	23	18	0
O_3	263	75	250	52
SO_2	171	34	63	6
CO	79	26	25	8
Mixture	104	29	30	6
**Analysis using the annual average exposures in previous year of blood draw as exposure indicators (*N* = 772)**				
$PM_{2.5}$	57	4	0	0
PM_{10}	134	27	26	3
NO_2	80	18	1	0
O_3	155	36	63	5
SO_2	162	40	77	3
CO	138	41	53	21
Mixture	75	8	1	0
**Analysis among controls (*N* = 528)**				
$PM_{2.5}$	61	10	0	0
PM_{10}	103	17	9	0
NO_2	79	10	0	0
O_3	140	25	21	1
SO_2	97	13	3	0
CO	110	14	2	0
Mixture	79	9	0	0
**Analysis among never-smokers (*N* = 606)**				
$PM_{2.5}$	44	7	0	0
PM_{10}	169	48	87	30
NO_2	66	10	0	0
O_3	236	73	184	49
SO_2	115	17	6	5
CO	87	15	1	0
Mixture	82	15	1	0

Note: $PM_{2.5}$, fine particulate matter; PM_{10}, coarse particulate matter; NO_2, nitrogen dioxide; O_3, ozone; SO_2, sulfur dioxide; CO, carbon monoxide. Mixture, air pollution mixture.

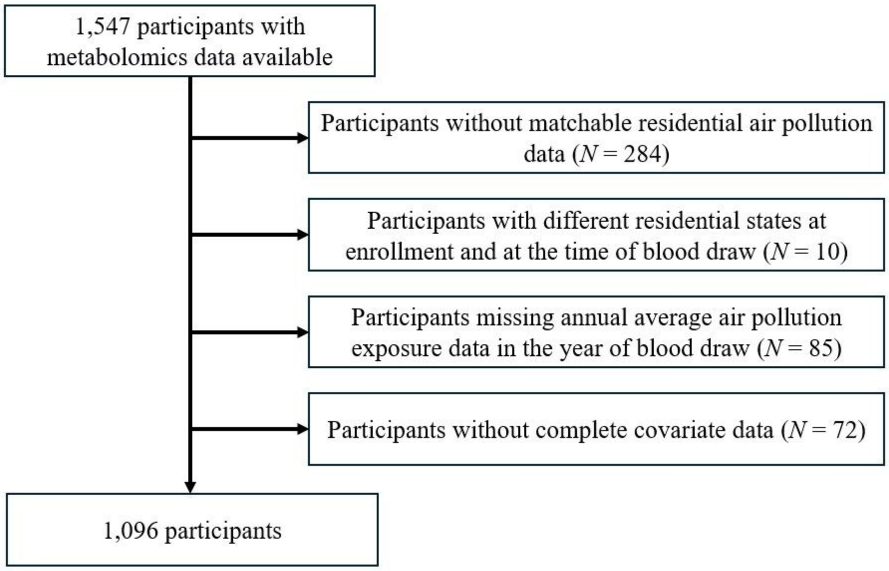

Figure S1. Flowchart of exclusion criteria for study participants in the final analysis.

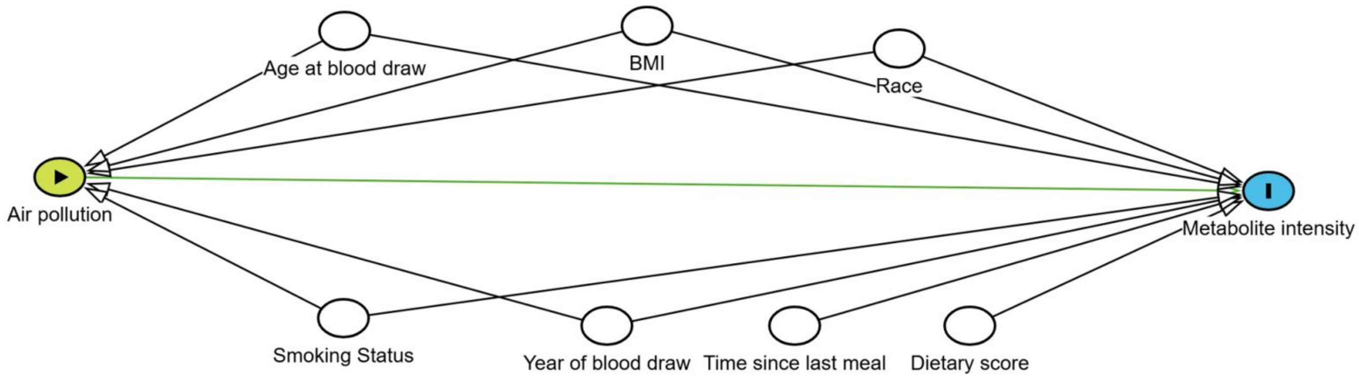

Figure S2. A directed acyclic graph of relationships among air pollution exposure, metabolite intensity, and covariates.

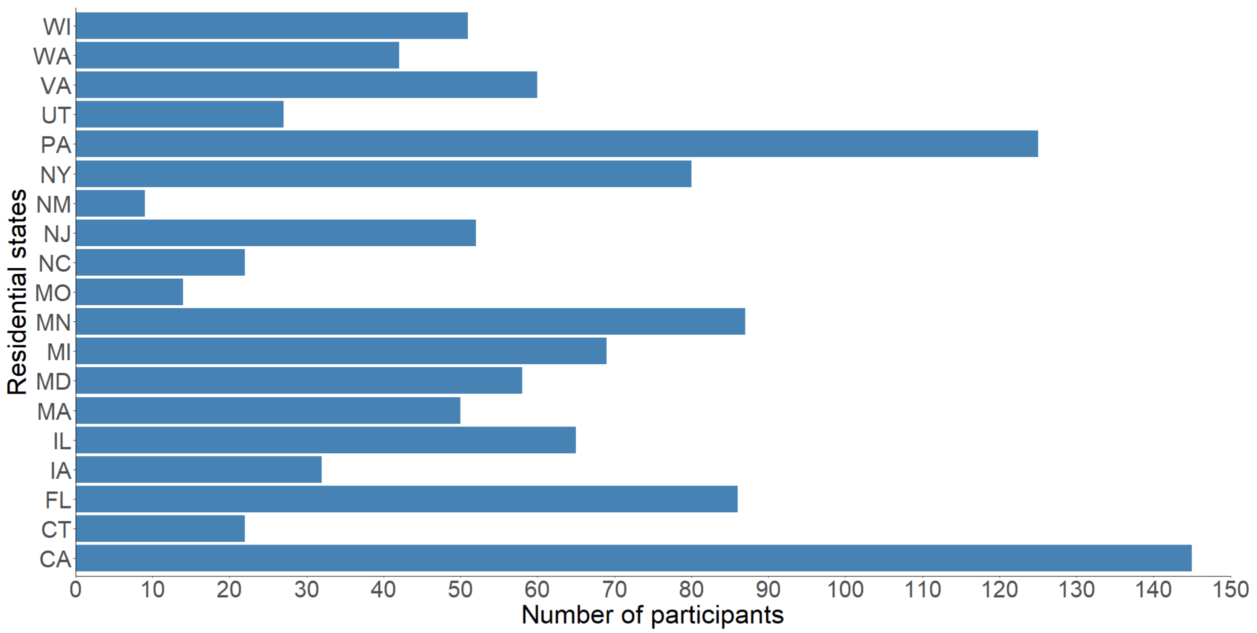

Figure S3. Distribution of residential states for the study population (*N* = 1,096).

Note: WI, Wisconsin; WA, Washington; VA, Virginia; UT, Utah; PA, Pennsylvania; NY, New York; NM, New Mexico; NJ, New Jersey; NC, North Carolina; MO, Missouri; MN, Minnesota; MI, Michigan; MD, Maryland; MA, Massachusetts; IL, Illinois; IA, Iowa; FL, Florida; CT, Connecticut; CA, California.

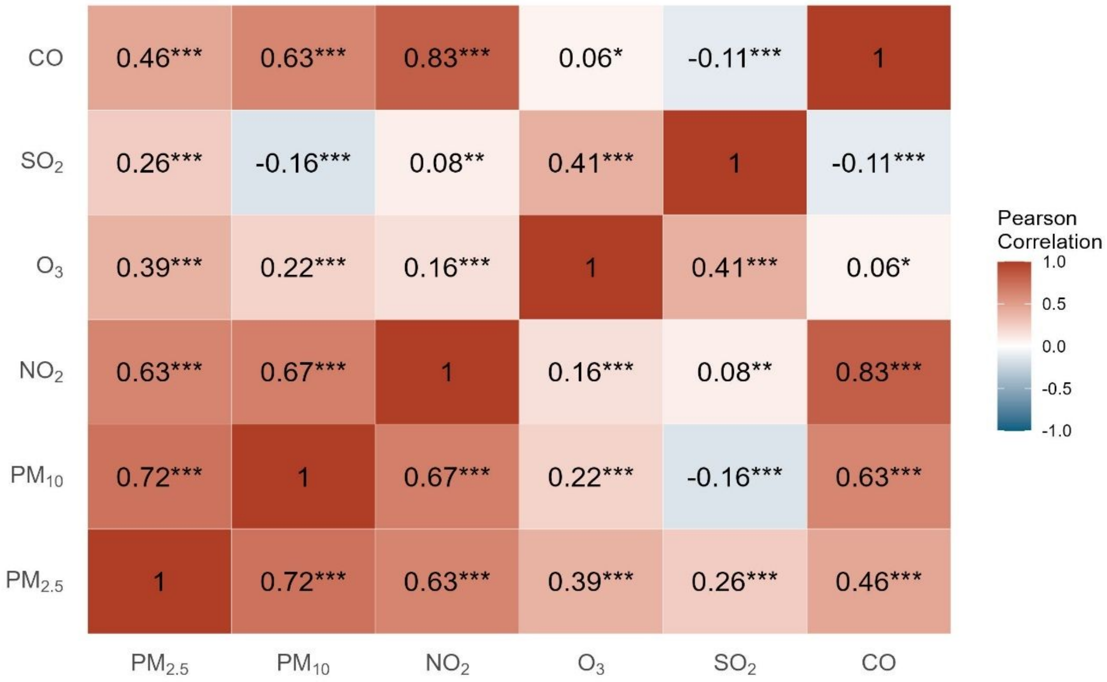

Figure S4. A heatmap of Pearson correlations among average exposure levels in the year of blood draw of six air pollutants.

Note: $PM_{2.5}$, fine particulate matter; PM_{10}, coarse particulate matter; NO_2, nitrogen dioxide; O_3, ozone; SO_2, sulfur dioxide; CO, carbon monoxide. For $PM_{2.5}$ and PM_{10}, the unit is $\mu g/m^3$; for NO_2, O_3, and SO_2, the unit is ppb; for CO, the unit is ppm.

Asterisks denote significance levels as follows: * $P < 0.05$; ** $P < 0.01$; *** $P < 0.001$.

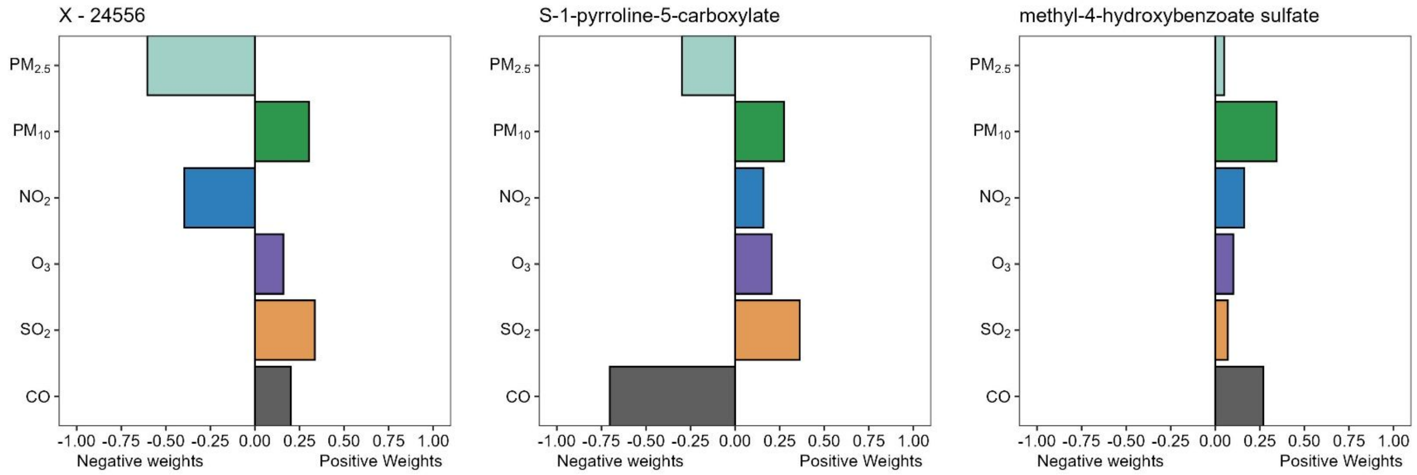

Figure S5. Weights of individual air pollutants for significant metabolites in air pollution mixture-metabolites models (FDR < 0.05). The weights represent the proportion of positive or negative partial effects on the overall mixture. The sum for positive weights is one, while the sum for negative weights is negative one. The positive and negative weights cannot be compared directly. The metabolites are ordered by FDR.

Note: $PM_{2.5}$, fine particulate matter; PM_{10}, coarse particulate matter; NO_2, nitrogen dioxide; O_3, ozone; SO_2, sulfur dioxide; CO, carbon monoxide; Mixture, air pollution mixture.

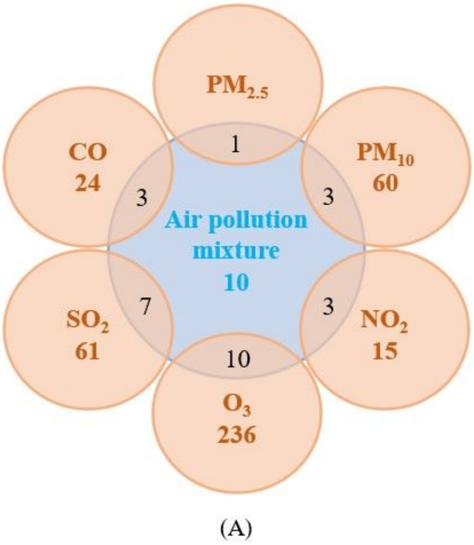

Figure S6. Overlapping metabolites associated with individual air pollutant exposure and air pollution mixture at (A) false discovery rate (FDR) < 0.2 and (B) unadjusted $P < 0.05$. The number in blue indicates the unique metabolites solely associated with the air pollution mixture. The number in orange indicates the unique metabolites solely associated with each air pollutant. The number in black indicates the overlapping metabolites associated with each air pollutant and air pollution mixture.

Note: $PM_{2.5}$, fine particulate matter; PM_{10}, coarse particulate matter; NO_2, nitrogen dioxide; O_3, ozone; SO_2, sulfur dioxide; CO, carbon monoxide.

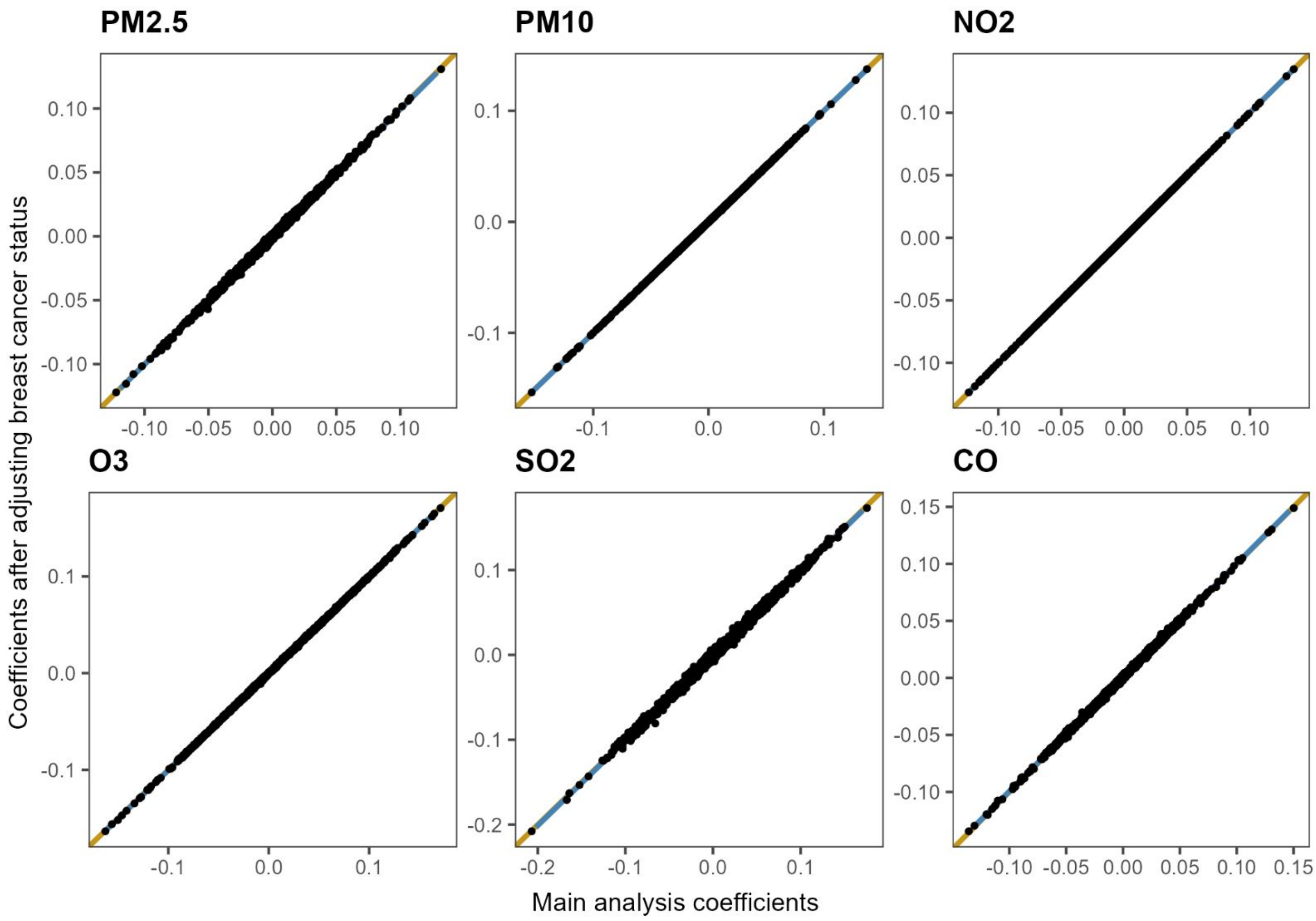

Figure S7. Relations of effect coefficients of metabolite-pollutants associations from metabolome-wide association study (MWAS) models before and after adjusting for breast cancer status. The main analysis adjusted for age at blood draw, body mass index, dietary score, race, smoking status, year of blood draw, and hours since last meal. Each black dot represents one metabolite. The blue line represents the linear regression line of all the dots. The yellow line represents the identity line (y=x).

Note: $PM_{2.5}$, fine particulate matter; PM_{10}, coarse particulate matter; NO_2, nitrogen dioxide; O_3, ozone; SO_2, sulfur dioxide; CO, carbon monoxide.

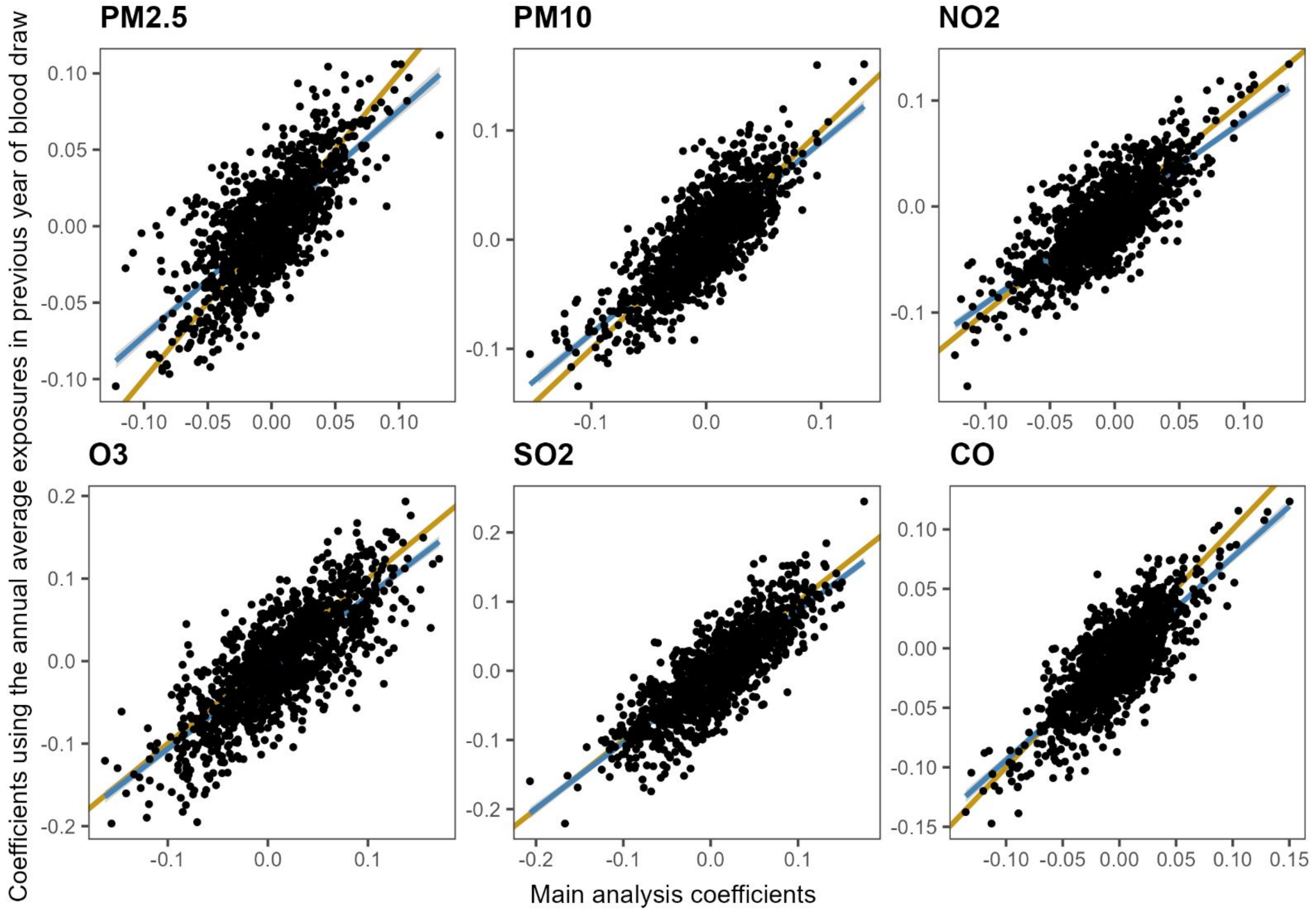

Figure S8. Relations of effect coefficients of metabolite-pollutants associations from metabolome-wide association study (MWAS) models between using annual average exposure in the current year of the blood draw and using annual average exposure in the previous year of the blood draw as exposure indicator. Both analyses were adjusted for age at blood draw, body mass index, dietary score, race, smoking status, year of blood draw, and hours since last meal. Each black dot represents one metabolite. The blue line represents the linear regression line of all the dots. The yellow line represents the identity line (y=x).

Note: $PM_{2.5}$, fine particulate matter; PM_{10}, coarse particulate matter; NO_2, nitrogen dioxide; O_3, ozone; SO_2, sulfur dioxide; CO, carbon monoxide.

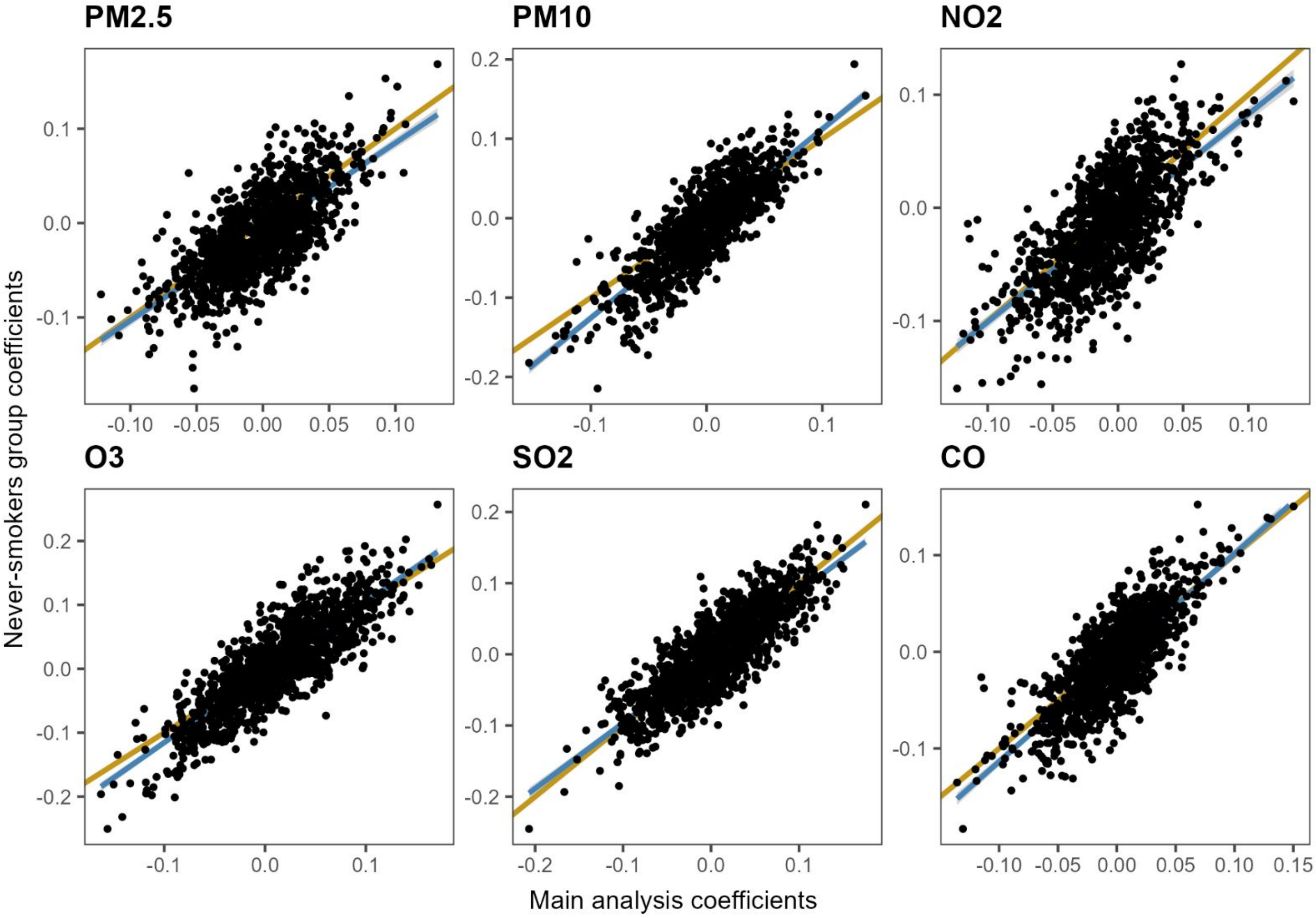

Figure S9. Relations of effect coefficients of metabolite-pollutants associations from metabolome-wide association study (MWAS) between the overall study population and among the never-smoker group. The main analysis was adjusted for age at blood draw, body mass index, dietary score, race, smoking status, year of blood draw, and hours since last meal. The analysis among the never-smoker group was adjusted for age at blood draw, body mass index, dietary score, race, year of blood draw, and hours since last meal. Each black dot represents one metabolite. The blue line represents the linear regression line of all the dots. The yellow line represents the identity line (y=x).

Note: $PM_{2.5}$, fine particulate matter; PM_{10}, coarse particulate matter; NO_2, nitrogen dioxide; O_3, ozone; SO_2, sulfur dioxide; CO, carbon monoxide.

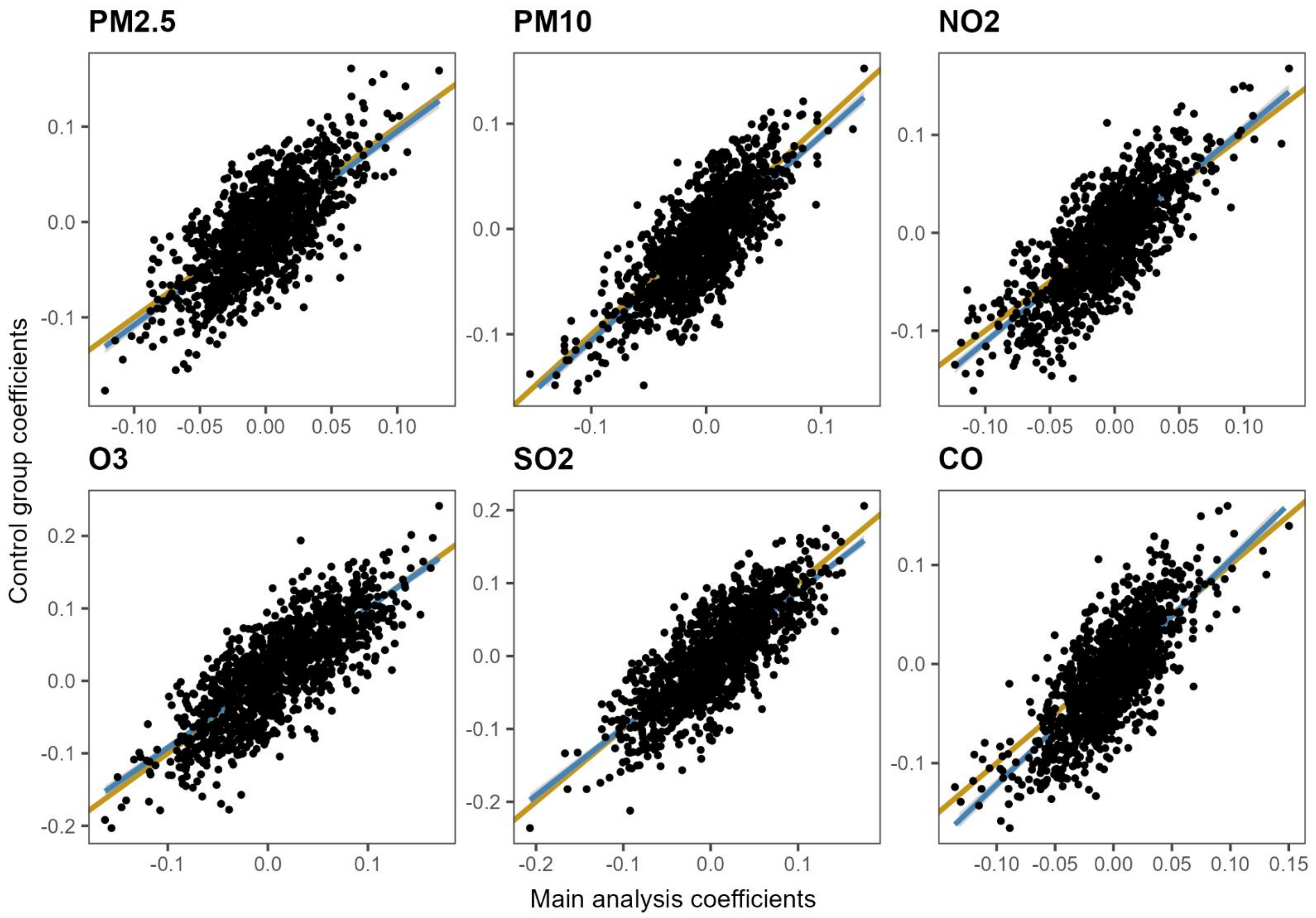

Figure S10. Relations of effect coefficients of metabolite-pollutants associations from metabolome-wide association study (MWAS) between the overall study population and among control group. Both analyses were adjusted for age at blood draw, body mass index, dietary score, race, smoking status, year of blood draw, and hours since last meal. Each black dot represents one metabolite. The blue line represents the linear regression line of all the dots. The yellow line represents the identity line (y=x).

Note: $PM_{2.5}$, fine particulate matter; PM_{10}, coarse particulate matter; NO_2, nitrogen dioxide; O_3, ozone; SO_2, sulfur dioxide; CO, carbon monoxide.

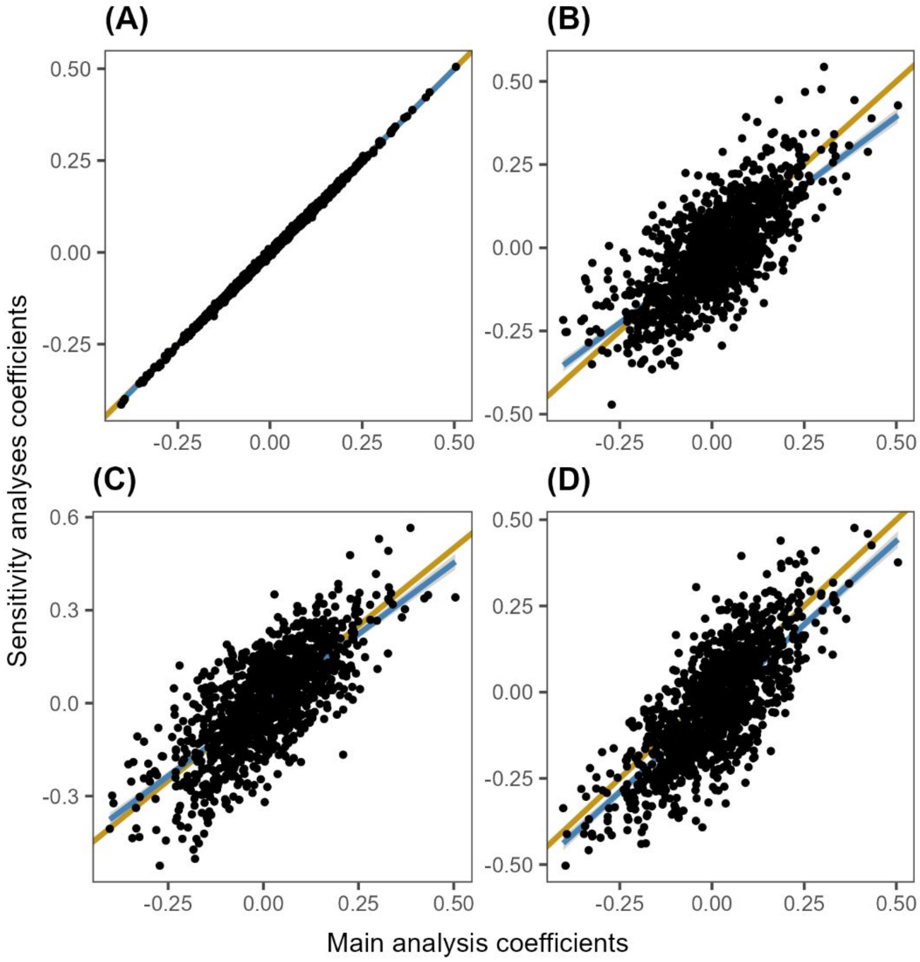

Figure S11. Relations of effect coefficients from air pollution mixture models (A) before and after adjusting for breast cancer status; (B) between using annual average exposure in the current year of the blood draw and using annual average exposure in the previous year of the blood draw as exposure indicator between the overall study population; (C) between the overall study population and among the never-smoker group; (D) between the overall study population and among control group. Each black dot represents one metabolite. The blue line represents the linear regression line of all the dots. The yellow line represents the identity line (y=x).
